# Hippocampal Offline Reactivation Consolidates Recently Formed Cell Assembly Patterns during Sharp Wave-Ripples

**DOI:** 10.1016/j.neuron.2016.10.020

**Published:** 2016-12-07

**Authors:** Gido M. van de Ven, Stéphanie Trouche, Colin G. McNamara, Kevin Allen, David Dupret

**Affiliations:** 1 MRC Brain Network Dynamics Unit, Department of Pharmacology, University of Oxford, OX1 3TH Oxford, UK; 2 Department of Clinical Neurobiology, Medical Faculty of Heidelberg University and DKFZ, 69120 Heidelberg, Germany

## Abstract

The ability to reinstate neuronal assemblies representing mnemonic information is thought to require their consolidation through offline reactivation during sleep/rest. To test this, we detected cell assembly patterns formed by repeated neuronal co-activations in the mouse hippocampus during exploration of spatial environments. We found that the reinstatement of assembly patterns representing a novel, but not a familiar, environment correlated with their offline reactivation and was impaired by closed-loop optogenetic disruption of sharp wave-ripple oscillations. Moreover, we discovered that reactivation was only required for the reinstatement of assembly patterns whose expression was gradually strengthened during encoding of a novel place. The context-dependent reinstatement of assembly patterns whose expression did not gain in strength beyond the first few minutes of spatial encoding was not dependent on reactivation. This demonstrates that the hippocampus can hold concurrent representations of space that markedly differ in their encoding dynamics and their dependence on offline reactivation for consolidation.

**Video Abstract:**

## Highlights

• Multi-neuron co-activations form space-coding assembly patterns in the hippocampus
• Offline reactivation of new assembly patterns correlates with future reinstatement
• SWR silencing impairs consolidation of patterns of a novel, but not a familiar, place
• “Early stabilized” patterns representing a novel place do not require reactivation

## Teaser

van de Ven et al. identify hippocampal cell assembly patterns formed by repeated multi-neuron co-activations and show that only those patterns that represent a novel place and that were gradually strengthened during their first expression require SWR reactivation for their context-dependent reinstatement.

## Introduction

Co-activation of groups of neurons forming cell assemblies is thought to underpin information representation in the brain (Hebb, 1949, Buzsáki, 2010). Within this framework, the ability to hold and retrieve newly formed assemblies allows the brain to store and recall previously encoded information. In the hippocampus, the firing of principal neurons is spatially tuned, and groups of co-active neurons can jointly represent discrete locations (O’Keefe and Dostrovsky, 1971, Wilson and McNaughton, 1993, Leutgeb et al., 2005). The extent to which the constellation of hippocampal assemblies representing an environment is later reinstated during context re-exposure could govern the ability to remember that environment (e.g., Kentros et al., 2004). In line with this, impaired reinstatement of hippocampal representations of space correlates with spatial memory deficits (Barnes et al., 1997).

Accumulating evidence suggests that new internal representations are stabilized by reactivating the underlying cell assemblies during the post-encoding sleep/rest period (Rasch and Born, 2007, O’Neill et al., 2010). Indeed, the joint firing of hippocampal neurons encoding nearby places during exploration recurs in subsequent sleep (Wilson and McNaughton, 1994). The related hypothesis is that repeated neuronal co-activation strengthens newly formed assemblies (Hebb, 1949). Offline reactivation is most prominent during sharp wave-ripple (SWR; 125–250 Hz) oscillatory events (Wilson and McNaughton, 1994, Buzsáki, 2015a) in which conditions indeed promote Hebbian synaptic plasticity (Sadowski et al., 2016). Consistent with a role for reactivation in memory consolidation, co-firing patterns associated with spatial novelty or rewarded learning are reactivated more strongly (O’Neill et al., 2008, Singer and Frank, 2009, McNamara et al., 2014), and electrical disruption of hippocampal SWRs during sleep impairs subsequent memory recall (Girardeau et al., 2009, Ego-Stengel and Wilson, 2010).

Despite an increasing number of studies advocating reactivation as a circuit-level mechanism for memory consolidation, a causal relation between the (sleep) reactivation of new assembly patterns and their subsequent (awake) reinstatement has not been demonstrated. Here, to test for a role of offline reactivation in the stabilization of neuronal traces of waking experiences, we identified in the mouse hippocampus assembly patterns formed by repeated neuronal co-firing during the first exploration of novel environments and tracked their expression strength during the following sleep/rest and context re-exposure. Using closed-loop optogenetic silencing of principal neurons, we then determined whether selective disruption of SWR reactivation during sleep/rest alters the future reinstatement of these patterns. Importantly, to test whether such a role of reactivation would be time limited, which is a defining criterion for a consolidation process (Dudai, 2004, Squire et al., 2015), we also detected, tracked, and SWR-silenced assembly patterns of a familiar environment. In doing so, we found that SWR reactivation is only required for the context-dependent reinstatement of hippocampal co-activation patterns representing a novel environment, and further discovered that the strengthening dynamic of new patterns during the initial encoding is predictive of their dependency on offline reactivation.

## Results

### Short-Timescale Co-activation of Hippocampal Neurons Forms Spatially Selective Assembly Patterns

**Figure 1:**
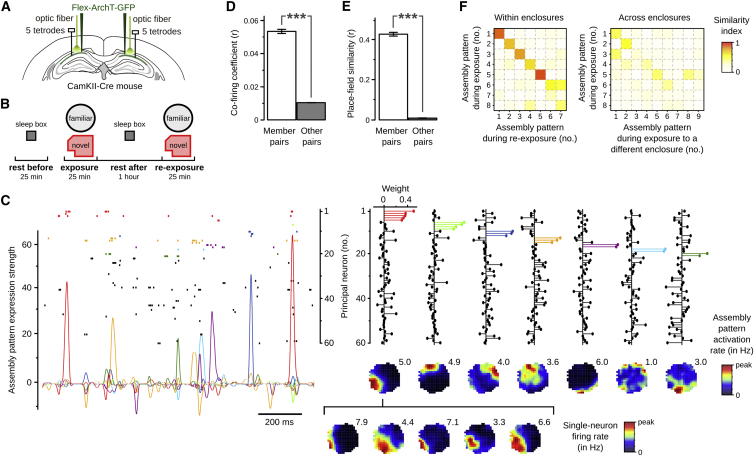
Short-Timescale Hippocampal Co-firing Patterns Are Spatially Tuned and Environment Specific (A) Ensemble recordings and optogenetic manipulation of CamKII::ArchT mice. (B) Schematic of one recording block, with repeated exposure to either the familiar or novel enclosure (see Figures S1A–S1D). (C) Assembly patterns identified from repeated coincident neuronal discharges in 25 ms time bins spanning the exposure session (see Figure S2). For visualization purposes, the 60 simultaneously recorded principal neurons are ordered and color coded to highlight neurons with high weight to the same pattern. Shown are an ∼1.5 s example raster plot of the spike trains (top left; one neuron per row), along with the expression strength time course of each detected pattern (bottom left), their weight vectors (top right), and corresponding assembly spatial maps (bottom right; numbers indicate peak assembly pattern activation rate). At the bottom, single-neuron firing rate maps (numbers indicate peak firing rate) are shown for the five neurons with high weight (highlighted in red) in the first pattern. (D and E) Detected assembly patterns group together neurons with correlated firing activity and overlapping spatial tuning. Both the average co-firing coefficient (D) and place-field similarity (E) are much higher for pairs of neurons with a high weight to the same pattern (n = 919 member pairs) than for other neuron pairs (n = 59,823 other pairs). Error bars represent ± 1 SEM; ^∗∗∗^p < 0.001. (F) Detected assembly patterns are environment specific. Example from one recording day showing that patterns expressed during an exposure session are more similar to those identified during re-exposure to that enclosure (left) than to those identified in another enclosure of a different recording block (right).

We monitored network activity from the dorsal CA1 hippocampus of CamKII-Cre mice (n = 8) using multichannel extracellular recordings (Figure 1A) during exploration of open-field enclosures alternating with periods of sleep/rest. Every day, principal neurons (44.4 ± 2.5 per day) were followed across multiple recording blocks (2.2 ± 0.1 per day; Figure 1B). During each block, mice explored either a novel or a familiar enclosure (“exposure”; Figures S1A–S1D, available online) and were re-exposed to that enclosure (“re-exposure”) after 1 hr in their sleep box (“sleep/rest”).

For each of the 93 recording blocks acquired, we aimed to identify groups of principal neurons with repeated coincident firing within short time windows during the exposure. We opted for 25 ms windows because it was previously suggested that neuronal co-activity at this timescale is optimal for cell assembly expression (Harris et al., 2003). A two-step statistical method first estimated the number of significant co-activation patterns in the spike trains and then extracted those patterns with an independent component analysis (Figure S2A; Lopes-dos-Santos et al., 2013). A total of 521 patterns (5.6 ± 0.2 per block) were identified, each described by a weight vector containing the contribution of each neuron (Figures 1C and S2A). We confirmed that pairs of neurons with a large contribution to the same “assembly pattern” had far stronger instantaneous rate correlations than other neuron pairs (Figure 1D).

We next assessed whether the detected assembly patterns carried behaviorally relevant information. When we tracked the expression of each pattern over time (Figure S2B), we found that their activations were spatially tuned (Figure 1C). In line with this, the discharge of neurons with a large contribution to the same pattern substantially overlapped in space (Figures 1C and 1E). Thus, although the assembly pattern detection was blind to the animal’s location and solely based on short-timescale co-activations, it successfully grouped together neurons representing the same location. However, the detection was not merely governed by the spatial overlap of neurons’ discharge, for distinct patterns of the same enclosure could overlap in space (e.g., the light green and orange patterns in Figure 1C). This suggests that a given location could be represented by several assembly patterns (Jackson and Redish, 2007).

We then applied this pattern detection method across the several recording blocks performed within each day to compare patterns expressed in distinct enclosures. We found that assembly patterns detected during exposure to a given enclosure were more similar to those detected during re-exposure to that same enclosure than to those detected in another enclosure on that day (Figure 1F; environment-specificity index, 0.18 ± 0.01; n = 237 patterns, p < 0.0001). Together, these results show that repeated short-timescale co-activations of hippocampal principal neurons form spatially selective assembly patterns that are reinstated upon context re-exposure.

### Disruption of Reactivation Impairs Reinstatement of Assembly Patterns Representing Novel, but Not Familiar, Environments

**Figure 2:**
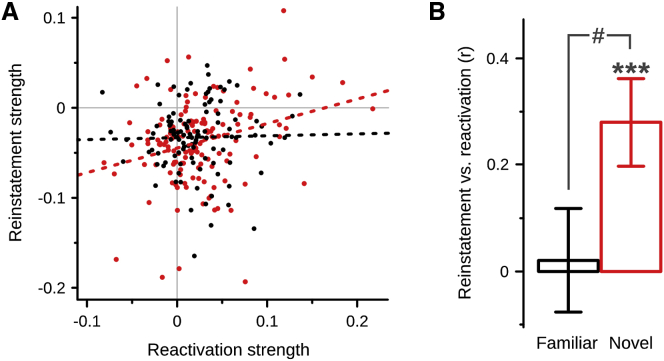
Assembly Pattern Reactivation Following a Novel, but Not Familiar, Enclosure Correlates with Upcoming Awake Reinstatement (A) Scatterplot of the reinstatement strength (change in expression strength from exposure to re-exposure) versus the reactivation strength (change in expression strength from rest before to rest after; see also Figures S1F–S1H) of assembly patterns detected in the familiar (black) or novel (red) enclosure. Dashed lines are corresponding ordinary least-squares regression lines (familiar, slope = 0.02, p = 0.81, R^2^ = 0.00; novel, slope = 0.27, p < 0.001, R^2^ = 0.08). (B) Correlation between reactivation and reinstatement strength is stronger after a novel enclosure than after the familiar one. Error bars represent ± 1 SE of the correlation coefficient; novel versus zero, ^∗∗∗^p < 0.001; familiar versus novel, ^#^p < 0.05.

We then checked whether the reinstatement of assembly patterns during context re-exposure correlated with their offline reactivation. To do so, the expression of assembly patterns identified during the exposure was tracked throughout each recording block (Figure 1B). We found that the average expression strength of 71.7% of these patterns was stronger during the rest following the exposure than during the rest preceding it (against chance, p < 0.0001; Figures S1F and S1G), confirming that waking assembly patterns were subsequently reactivated in the sleep box. Importantly, the reactivation strength of patterns expressed in novel enclosures, but not in the familiar one, correlated with their reinstatement strength during context re-exposure (Figure 2).

**Figure 3:**
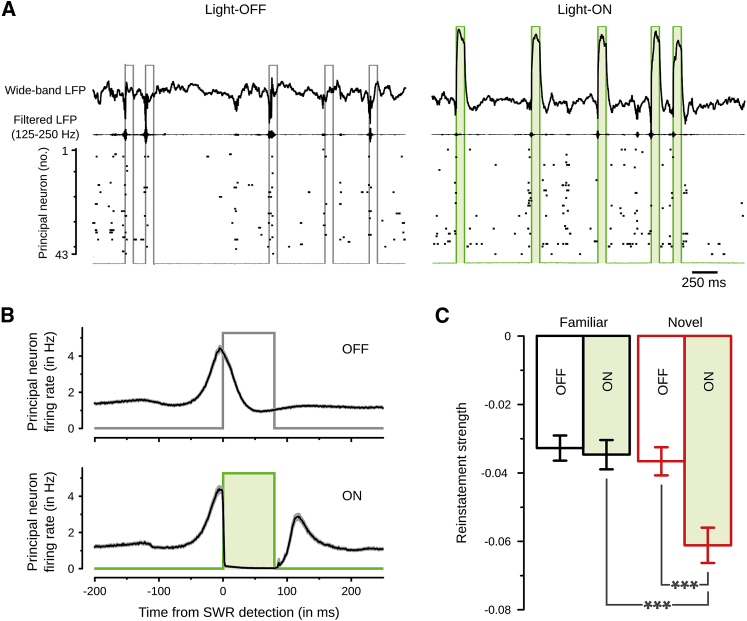
Optogenetic SWR Silencing Impairs Reinstatement of Assembly Patterns Associated with a Novel, but Not Familiar, Enclosure (A and B) Closed-loop feedback transiently silencing principal neurons during SWRs is illustrated with a raw data example (A) and quantified by the firing rate response (mean ± SEM) of principal neurons (B; light-OFF, n = 1,988 neurons; light-ON, n = 1,527). (C) After exposure to a novel enclosure, SWR silencing impairs the reinstatement of assembly patterns during context re-exposure (light-OFF, n = 139 patterns; light-ON, n = 136). This is not the case following exposure to the familiar enclosure (light-OFF, n = 108 patterns; light-ON, n = 78). As reinstatement strength is defined by the change in a pattern’s average expression strength from exposure to re-exposure, a null score corresponds to “perfect” reinstatement while the more negative, the worse the reinstatement. Data are represented as mean ± SEM; ^∗∗∗^p < 0.001. See also Figure S3.

The reactivation of assembly patterns was strongest during SWRs (Figure S1H), in line with studies based on pairwise correlations (Wilson and McNaughton, 1994, O’Neill et al., 2008). Therefore, to test whether offline reactivation of assembly patterns is required for their subsequent awake reinstatement, we performed closed-loop optogenetic silencing of principal neurons during SWRs (Figure S3A). We injected the dorsal CA1 hippocampus of CamKII-Cre mice (n = 7) with a flex-ArchT-GFP viral construct to target principal neurons with the light-driven proton pump ArchT (Figure S3B). Mice were then implanted with tetrodes and optic fibers to monitor and manipulate neuronal discharge (Figure 1A). In rest sessions without light delivery, 80.1% ± 1.0% of the SWRs were detected in real time with an average latency of 7.68 ± 0.30 ms before their peak power. When light was delivered upon SWR detection, principal neurons were silenced within 3.07 ± 0.54 ms from the light onset and returned to baseline firing within 22.12 ± 1.01 ms following the light offset (Figures 3A, 3B, and S3C). We found that SWR silencing applied during rest following a novel enclosure impaired assembly pattern reinstatement during context re-exposure (Figures 3C, S3E, and S3F). Importantly, the same SWR silencing during rest following the familiar enclosure did not alter the reinstatement of its assembly patterns (Figure 3C; with interaction SWR-silencing x enclosure type, F(1,318) = 5.05, p < 0.05). Moreover, after random optogenetic silencing performed independently of SWR occurrence, patterns expressed in a novel enclosure were reinstated stronger than after SWR silencing (Figures S3C–S3E). These results, further confirmed using conventional neuron-pair and single-neuron analyses (Figures S3G and S3H), establish that offline reactivation during SWRs is required to stabilize newly expressed co-activation patterns.

### Gradually Strengthened, but Not Early Stabilized, Assembly Patterns Require Reactivation

**Figure 4:**
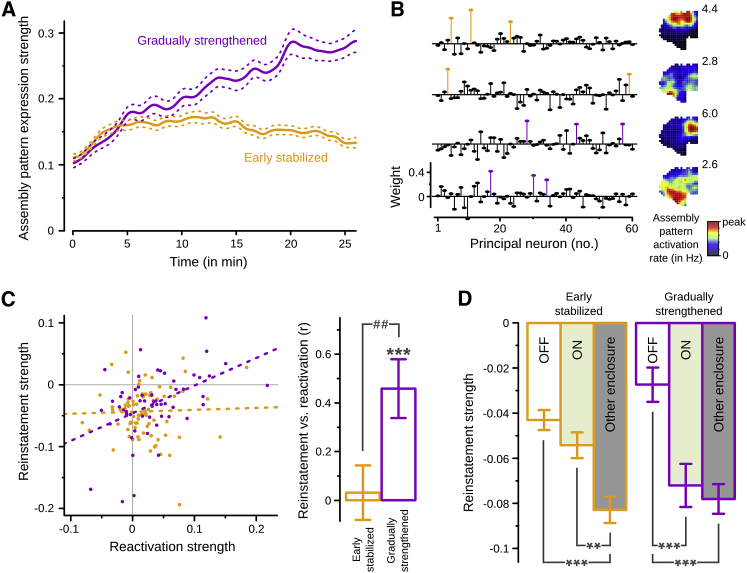
Offline Reactivation Is Required for Reinstatement of Gradually Strengthened, but Not of Early Stabilized, Assembly Patterns (A) The expression strength (mean ± SEM) of gradually strengthened patterns (purple; n = 134) continually increases during the exposure session, while that of early stabilized patterns (orange; n = 201) is more stable. Yet both sets are equally strengthened in the first few minutes. (B) Examples of two early stabilized (top) assembly patterns simultaneously expressed with two gradually strengthened (bottom) assembly patterns. (C) In the light-OFF condition, the reactivation of gradually strengthened patterns, but not of early stabilized ones, correlates with their reinstatement strength during context re-exposure. Dashed lines are corresponding ordinary least-squares regression lines (early stabilized, slope = 0.03, p = 0.78, R^2^ = 0.00; gradually strengthened, slope = 0.45, p < 0.001, R^2^ = 0.21). Error bars represent ± 1 SE of the correlation coefficient; gradually strengthened versus zero, ^∗∗∗^p < 0.001; early stabilized versus gradually strengthened, ^##^p < 0.01. (D) SWR silencing does not impair the context-dependent reinstatement of early stabilized patterns (light-OFF, n = 82 patterns; light-ON, n = 83; other enclosure, n = 155), but causes the reinstatement of gradually strengthened patterns to drop to the unspecific level at which they are expressed in a distinct enclosure of another recording block that day (light-OFF, n = 57 patterns; light-ON, n = 53; other enclosure, n = 103). Data are represented as mean ± SEM; ^∗∗^p < 0.01, ^∗∗∗^p < 0.001. See also Table S1.

If repeated neuronal co-activation strengthens a newly formed assembly (Hebb, 1949), then the strength of the corresponding firing pattern would be expected to increase throughout its formation. To test for such a strengthening dynamic, we fitted a linear trend to the expression strength of each assembly pattern during the first exposure to a novel enclosure. We found a significant positive slope for 134 out of 335 patterns (40.0%), compared to only 18 patterns (5.4%) with a significant negative slope. We refer to the patterns with a significant increasing linear trend as “gradually strengthened” (Figure 4A). Interestingly, the remaining patterns showed a similar strengthening only during the first few minutes (Figure 4A). This initial positive trend could reflect the rapid recruitment of these patterns during the first exposure to an enclosure, and we refer to them as “early stabilized.” Importantly, gradually strengthened and early stabilized patterns had similar composition of their weight vectors and were equally spatially selective (Figure 4B; Table S1).

We finally tested whether these concurrently expressed assembly patterns equally required offline reactivation for their lasting expression. Both sets of patterns were reactivated; the reactivation of the gradually strengthened patterns was stronger (Table S1). Importantly, only the reactivation strength of the gradually strengthened patterns, and not of the early stabilized ones, correlated with their future reinstatement during context re-exposure (Figure 4C). Moreover, SWR silencing only impaired the reinstatement of the gradually strengthened patterns (Figure 4D; with interaction SWR-silencing x pattern type, F(1,271) = 6.28, p < 0.05). In the baseline condition (i.e., no optogenetic silencing), the reinstatement of both early stabilized and gradually strengthened patterns was context dependent (Figure 4D; “light-OFF” versus “other enclosure”). SWR silencing decreased the reinstatement of gradually strengthened patterns down to their non-specific strength level seen in a different enclosure, but it did not significantly affect the reinstatement of the early stabilized patterns (Figure 4D; “light-ON” versus “other enclosure”).

## Discussion

Our study establishes that the context-dependent reinstatement of hippocampal co-firing patterns requires SWR reactivation following their initial expression during spatial exploration. The idea that the stabilization of newly formed cell assembly patterns involves their reactivation during resting behavior has been a long-standing hypothesis central to many theories of memory consolidation, although it has never been directly tested. Here, by combining ensemble recordings with an unsupervised statistical framework, we identified short-timescale co-activation patterns of CA1 principal neurons, which we showed to be spatially selective. We observed that for only a specific set of these patterns, those with continued strengthening throughout their initial expression, the reinstatement during context re-exposure was both correlated with their reactivation and suppressed by optogenetic SWR silencing. This study therefore provides direct evidence that the stabilization of recently formed, space-representing hippocampal cell assembly patterns depends on offline reactivation.

### Time-Limited Role of SWR Reactivation in the Persistence of Neuronal Representations of Space Could Underlie Memory Consolidation

Previous studies showed that post-learning disruption of sleep SWRs by electrical stimulation of the ventral hippocampal commissure impaired spatial memory performance (Girardeau et al., 2009, Ego-Stengel and Wilson, 2010), thereby laying the foundation for an instrumental role of SWRs in memory. However, it was not possible in these studies to establish whether the observed impairment was caused by the disruption of the SWR-associated reactivation of waking firing patterns, or due to an unspecific effect of electrical stimulation coupled to SWRs. Moreover, it remained to be tested whether the effect of SWR disruption depends on such a manipulation being applied shortly after encoding. Indeed, to decisively demonstrate that a process has a role in consolidation, it is required to show that its disruption has a time-limited effect, namely that its disruption affects the persistence of traces of recent experiences and not those of remote ones (Dudai, 2004, Squire et al., 2015).

Here, we directly silenced SWR reactivation using an optogenetic approach. We found that this intervention disrupted the upcoming reinstatement of hippocampal assembly patterns when performed after the first exploration of a (thus novel) environment. Importantly, the same SWR silencing was ineffective on pattern reinstatement when performed after an environment that had been repetitively experienced before (hence familiar). This control condition rules out a generic effect of SWR disruption. Indeed, the familiar environment is here a “delayed-block condition” that establishes the time-limited role of SWR reactivation. Our results, combined with the previously demonstrated behavioral effects, provide converging evidence that SWR reactivation supports memory consolidation by stabilizing the underlying cell assemblies.

The lack of effect of SWR silencing on the reinstatement of “familiar” assembly patterns raises the question of why these patterns are still reactivated. One explanation could be that the repetitive explorations of a given environment lead to the formation of multiple “entry points” to the same assemblies. This is reminiscent of the idea that re-experiencing a given memory is associated with the formation of multiple neuronal traces (Moscovitch et al., 2006). In this scenario, reactivation following exploration of the familiar environment might still stabilize some of these additional traces, but SWR silencing is ineffective because previously stabilized traces are sufficient to retrieve the assembly patterns representing that environment. Another possibility is that reactivation no longer stabilizes “familiar” patterns within the hippocampus, but still contributes to their “transfer” to downstream circuits (Squire et al., 2015).

### Early Stabilized versus Gradually Strengthened Assembly Patterns

Our study shows that for those assembly patterns that had an increasing expression strength over continued experience in a novel environment, their subsequent reinstatement was correlated with their reactivation and disrupted by SWR silencing. The features of this set of patterns are consistent with the Hebbian postulate of “fire together, wire together.” Conversely, the reinstatement of the other, concurrently expressed patterns that were no longer strengthened after the first few minutes of exploration was not correlated with their reactivation and unaffected by SWR silencing. Yet both sets were equally spatially selective and thus appeared to carry a similar representational attribute. These findings indicate that concurrent space-representing assembly patterns can markedly differ in their plastic properties.

The early stabilized patterns might have gained independence from offline reactivation because they rapidly acquired the status of “familiar” patterns while stably expressed during the exposure session. In this scenario, their consolidation would take place “on line” and the inefficacy of SWR silencing on these patterns would be an extreme reflection of the time-limited role of SWR reactivation. Perhaps, early stabilized patterns could be quickly consolidated because the place fields of their contributing neurons remap more coherently in the novel enclosure, for example, according to a topographical transform, rather than unpredictably (cf. Figure S1E). Another, non-exclusive possibility is that the early stabilized patterns are more “hardwired” to represent the new spatial layout due to the specifics of their contributing neurons in terms of existing spatial inputs or intrinsic properties. The gradually strengthened patterns, in contrast, could gain their spatial selectivity and increased strength by more plastic changes throughout the exploration. Under this scenario, the difference in strength between both sets of patterns could reflect that such plasticity enables neurons to better synchronize with their peers. An interesting related hypothesis is that the early stabilized patterns could provide a “nearly automatic” and yet stable representation of space, “ready to use” by downstream circuits, for instance, for (immediate) navigational purposes. As the animal accumulates experiences in the environment, the strengthening of the gradually strengthened patterns could reflect the formation of additional, perhaps richer, memory traces (Buzsáki, 2015b, Schiller et al., 2015).

### Conclusions

Altogether, this study establishes that the lasting expression of recently formed hippocampal co-activation patterns that resemble classical Hebbian assemblies requires their offline reactivation. Our findings support the long-standing hypothesis of an instrumental role of offline SWR reactivation in the consolidation of memory-representing assemblies. However, reactivation-dependent assembly patterns were co-expressed with other space-coding patterns that did not require offline reactivation. As a pattern’s dependency on offline SWR reactivation was related to its strengthening dynamics during spatial encoding, this study therefore highlights functional heterogeneity within co-expressed representations of space.

## Experimental Procedures

Full details of the procedures are provided in the Supplemental Experimental Procedures.

### Animals, Ensemble Recordings, and Optogenetic Silencing

All animals used were male adult transgenic CamKIIa-Cre mice (RRID: IMSR_JAX:005359). To silence principal neurons, mice were injected with a Cre-dependent ArchT-GFP viral vector into the dorsal CA1 hippocampus. CamKII::ArchT mice were then implanted with ten tetrodes combined with two optic fibers to monitor and manipulate the activity of CA1 principal neurons (Figures 1A and S4). Each mouse performed multiple recording blocks (Figure 1B) per day. Every day, the animal was first recorded in its sleep box (“rest before”; ∼25 min). For each recording block, the animal was then allowed to successively explore an open-field enclosure (“exposure”; ∼25 min), rest for 1 hr in its sleep box (“rest after”), and again explore the same enclosure (“re-exposure”; ∼25 min). The open field was either familiar (i.e., repeatedly explored prior to the recordings) or novel (i.e., never seen before). In some recording blocks, optogenetic SWR silencing was performed during the “rest after.” For this, SWRs were detected in real time using the ripple-frequency band power to trigger delivery of a 561 nm light pulse (Figure S3A). In some other blocks, random silencing was instead performed, with a matched number of light pulses delivered independently of SWRs. All experiments involving animals were conducted according to the UK Animals (Scientific Procedures) Act 1986 under personal and project licenses issued by the Home Office following ethical review.

### Assembly Pattern Analysis

Neuronal co-firing patterns were detected using an unsupervised statistical framework based on independent component analysis. Spikes of each principal neuron were counted in 25 ms time bins covering the exposure session. To avoid a bias toward neurons with higher firing rates, the binned spike counts were z scored. Assembly patterns were then extracted in a two-step procedure (Figure S2A). First, the number of significant co-activation patterns embedded within the dataset was estimated as the number of principal component variances above a threshold derived from an analytical probability function for uncorrelated data (Marčenko-Pastur distribution). Then, an independent component analysis extracted the assembly patterns from the projection of the data into the subspace spanned by these significant principal components.

To track the expression of these assembly patterns over time (Figure S2B), a projection matrix was constructed for each pattern from the outer product of its weight vector. This allowed the computation of the similarity between each pattern and the recorded firing activity at any given time. The main diagonal of the projection matrix was set to zero to ensure that only co-activations of at least two neurons could contribute to the expression of a pattern. To achieve a high temporal resolution, the spike train of each neuron was convolved with a Gaussian kernel (and then z scored). The expression strength of a pattern at any point in time was then defined as the quadratic form of its projection matrix with the smoothed and z scored firing rate vector. The assembly pattern activations used to compute assembly maps were defined as peaks in the expression strength above 5. Note that each detected pattern had many of such activations over time (average activation rate = 0.95 ± 0.02 Hz). For each pattern detected during the exposure, its reactivation strength was defined as the difference in its average expression strength during “rest after” minus that during “rest before.” Its reinstatement strength was similarly defined as the difference in its average expression strength during “re-exposure” minus that during the “exposure.” Patterns detected in a novel enclosure were classified as gradually strengthened if a significant positive linear trend could be fitted to their expression strength during the exposure, and as early stabilized otherwise.

### Statistical Analysis

Details of all performed statistical tests are provided in the Supplemental Experimental Procedures. All tests based on a test statistic with a symmetric distribution were performed two sided. Reported group data are mean ± SEM, unless stated otherwise.

## Author Contributions

Conceptualization, G.M.v.d.V. and D.D.; Methodology, G.M.v.d.V. and D.D.; Software, G.M.v.d.V., C.G.M., K.A., and D.D.; Formal Analysis, G.M.v.d.V.; Investigation, G.M.v.d.V. and S.T.; Writing – Original Draft, G.M.v.d.V. and D.D.; Writing – Reviewing & Editing, G.M.v.d.V., S.T., C.G.M., K.A., and D.D.; Visualization, G.M.v.d.V.; Supervision, D.D.; Funding Acquisition, D.D.

## Supplemental Information

Document S1. Supplemental Experimental Procedures, Figures S1–S4, and Table S1

Document S2. Article plus Supplemental Information
