# Supplementary figures and images for: Hippocampal Offline Reactivation Consolidates Recently Formed Cell Assembly Patterns during Sharp Wave-Ripples
Source: Neuron. 2016 Dec 7;92(5):968–74. doi: 10.1016/j.neuron.2016.10.020 (PMC5158132; doi:10.1016/j.neuron.2016.10.020)

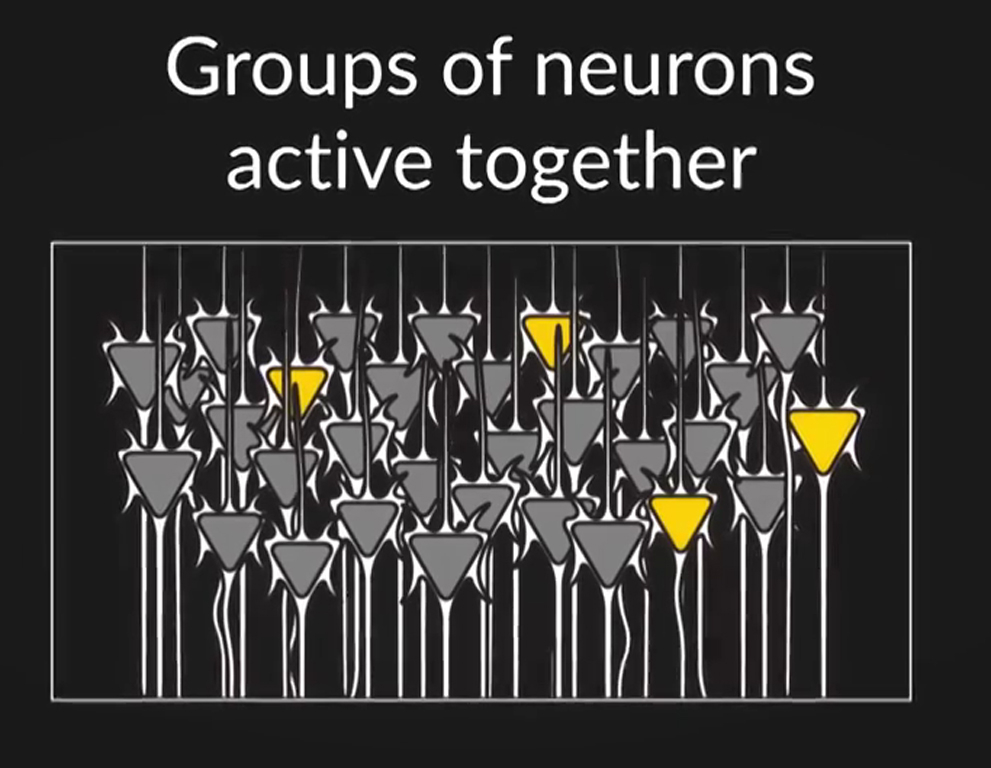

Supplement: Supplementary file 1 [file mmc3.jpg]
