# Supplementary material for: Hippocampal Offline Reactivation Consolidates Recently Formed Cell Assembly Patterns during Sharp Wave-Ripples
Source: Neuron. 2016 Dec 7;92(5):968–74. doi: 10.1016/j.neuron.2016.10.020 (PMC5158132; doi:10.1016/j.neuron.2016.10.020)
Supplement: Document S2. Article plus Supplemental Information [file mmc2.pdf]

Neuron

Report

Hippocampal Offline Reactivation Consolidates Recently Formed Cell Assembly Patterns during Sharp Wave-Ripples

Highlights

- Multi-neuron co-activations form space-coding assembly patterns in the hippocampus
- Offline reactivation of new assembly patterns correlates with future reinstatement
- SWR silencing impairs consolidation of patterns of a novel, but not a familiar, place
- “Early stabilized” patterns representing a novel place do not require reactivation

Authors

Gido M. van de Ven,
Stéphanie Trouche,
Colin G. McNamara, Kevin Allen,
David Dupret

Correspondence

gido.vandeven@pharm.ox.ac.uk
(G.M.v.d.V.),
david.dupret@pharm.ox.ac.uk (D.D.)

In Brief

van de Ven et al. identify hippocampal cell assembly patterns formed by repeated multi-neuron co-activations and show that only those patterns that represent a novel place and that were gradually strengthened during their first expression require SWR reactivation for their context-dependent reinstatement.

CrossMark

van de Ven et al., 2016, Neuron *92*, 968–974
December 7, 2016 © 2016 The Authors. Published by Elsevier Inc.
http://dx.doi.org/10.1016/j.neuron.2016.10.020

CellPress

Neuron
Report

Hippocampal Offline Reactivation Consolidates Recently Formed Cell Assembly Patterns during Sharp Wave-Ripples

Gido M. van de Ven,[1,*] Stéphanie Trouche,[1] Colin G. McNamara,[1] Kevin Allen,[2] and David Dupret[1,3,*]
[1]MRC Brain Network Dynamics Unit, Department of Pharmacology, University of Oxford, OX1 3TH Oxford, UK
[2]Department of Clinical Neurobiology, Medical Faculty of Heidelberg University and DKFZ, 69120 Heidelberg, Germany
[3]Lead Contact
*Correspondence: gido.vandeven@pharm.ox.ac.uk (G.M.v.d.V.), david.dupret@pharm.ox.ac.uk (D.D.)
http://dx.doi.org/10.1016/j.neuron.2016.10.020

SUMMARY

The ability to reinstate neuronal assemblies representing mnemonic information is thought to require their consolidation through offline reactivation during sleep/rest. To test this, we detected cell assembly patterns formed by repeated neuronal co-activations in the mouse hippocampus during exploration of spatial environments. We found that the reinstatement of assembly patterns representing a novel, but not a familiar, environment correlated with their offline reactivation and was impaired by closed-loop optogenetic disruption of sharp wave-ripple oscillations. Moreover, we discovered that reactivation was only required for the reinstatement of assembly patterns whose expression was gradually strengthened during encoding of a novel place. The context-dependent reinstatement of assembly patterns whose expression did not gain in strength beyond the first few minutes of spatial encoding was not dependent on reactivation. This demonstrates that the hippocampus can hold concurrent representations of space that markedly differ in their encoding dynamics and their dependence on offline reactivation for consolidation.

INTRODUCTION

Co-activation of groups of neurons forming cell assemblies is thought to underpin information representation in the brain (Hebb, 1949; Buzsáki, 2010). Within this framework, the ability to hold and retrieve newly formed assemblies allows the brain to store and recall previously encoded information. In the hippocampus, the firing of principal neurons is spatially tuned, and groups of co-active neurons can jointly represent discrete locations (O'Keefe and Dostrovsky, 1971; Wilson and McNaughton, 1993; Leutgeb et al., 2005). The extent to which the constellation of hippocampal assemblies representing an environment is later reinstated during context re-exposure could govern the ability to remember that environment (e.g., Kentros et al., 2004). In line with this, impaired reinstatement of hippocampal representations of space correlates with spatial memory deficits (Barnes et al., 1997).

Accumulating evidence suggests that new internal representations are stabilized by reactivating the underlying cell assemblies during the post-encoding sleep/rest period (Rasch and Born, 2007; O'Neill et al., 2010). Indeed, the joint firing of hippocampal neurons encoding nearby places during exploration recurs in subsequent sleep (Wilson and McNaughton, 1994). The related hypothesis is that repeated neuronal co-activation strengthens newly formed assemblies (Hebb, 1949). Offline reactivation is most prominent during sharp wave-ripple (SWR; 125–250 Hz) oscillatory events (Wilson and McNaughton, 1994; Buzsáki, 2015a) in which conditions indeed promote Hebbian synaptic plasticity (Sadowski et al., 2016). Consistent with a role for reactivation in memory consolidation, co-firing patterns associated with spatial novelty or rewarded learning are reactivated more strongly (O'Neill et al., 2008; Singer and Frank, 2009; McNamara et al., 2014), and electrical disruption of hippocampal SWRs during sleep impairs subsequent memory recall (Girardeau et al., 2009; Ego-Stengel and Wilson, 2010).

Despite an increasing number of studies advocating reactivation as a circuit-level mechanism for memory consolidation, a causal relation between the (sleep) reactivation of new assembly patterns and their subsequent (awake) reinstatement has not been demonstrated. Here, to test for a role of offline reactivation in the stabilization of neuronal traces of waking experiences, we identified in the mouse hippocampus assembly patterns formed by repeated neuronal co-firing during the first exploration of novel environments and tracked their expression strength during the following sleep/rest and context re-exposure. Using closed-loop optogenetic silencing of principal neurons, we then determined whether selective disruption of SWR reactivation during sleep/rest alters the future reinstatement of these patterns. Importantly, to test whether such a role of reactivation would be time limited, which is a defining criterion for a consolidation process (Dudai, 2004; Squire et al., 2015), we also detected, tracked, and SWR-silenced assembly patterns of a familiar environment. In doing so, we found that SWR reactivation is only required for the context-dependent reinstatement of hippocampal co-activation patterns representing a novel environment, and further discovered that the strengthening dynamic of new patterns during the initial encoding is predictive of their dependency on offline reactivation.

 © 2016 The Authors. Published by Elsevier Inc.
This is an open access article under the CC BY license (http://creativecommons.org/licenses/by/4.0/).

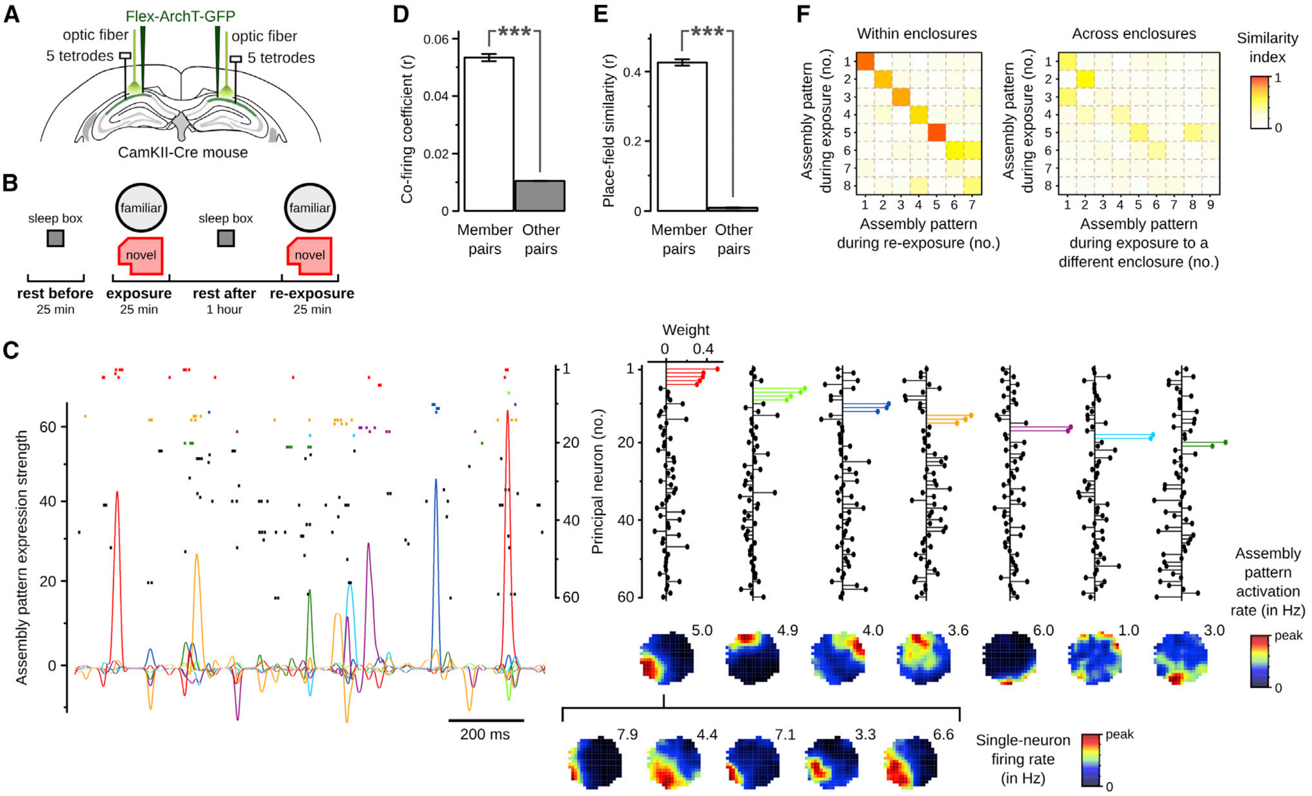

Figure 1. Short-Timescale Hippocampal Co-firing Patterns Are Spatially Tuned and Environment Specific

(A) Ensemble recordings and optogenetic manipulation of CamKII::ArchT mice.

(B) Schematic of one recording block, with repeated exposure to either the familiar or novel enclosure (see Figures S1A–S1D).

(C) Assembly patterns identified from repeated coincident neuronal discharges in 25 ms time bins spanning the exposure session (see Figure S2). For visualization purposes, the 60 simultaneously recorded principal neurons are ordered and color coded to highlight neurons with high weight to the same pattern. Shown are an ~1.5 s example raster plot of the spike trains (top left; one neuron per row), along with the expression strength time course of each detected pattern (bottom left), their weight vectors (top right), and corresponding assembly spatial maps (bottom right; numbers indicate peak assembly pattern activation rate). At the bottom, single-neuron firing rate maps (numbers indicate peak firing rate) are shown for the five neurons with high weight (highlighted in red) in the first pattern.

(D and E) Detected assembly patterns group together neurons with correlated firing activity and overlapping spatial tuning. Both the average co-firing coefficient (D) and place-field similarity (E) are much higher for pairs of neurons with a high weight to the same pattern (n = 919 member pairs) than for other neuron pairs (n = 59,823 other pairs). Error bars represent ± 1 SEM; ***p < 0.001.

(F) Detected assembly patterns are environment specific. Example from one recording day showing that patterns expressed during an exposure session are more similar to those identified during re-exposure to that enclosure (left) than to those identified in another enclosure of a different recording block (right).

RESULTS

Short-Timescale Co-activation of Hippocampal Neurons Forms Spatially Selective Assembly Patterns

We monitored network activity from the dorsal CA1 hippocampus of CamKII-Cre mice (n = 8) using multichannel extracellular recordings (Figure 1A) during exploration of open-field enclosures alternating with periods of sleep/rest. Every day, principal neurons (44.4 ± 2.5 per day) were followed across multiple recording blocks (2.2 ± 0.1 per day; Figure 1B). During each block, mice explored either a novel or a familiar enclosure (“exposure”; Figures S1A–S1D, available online) and were re-exposed to that enclosure (“re-exposure”) after 1 hr in their sleep box (“sleep/rest”).

For each of the 93 recording blocks acquired, we aimed to identify groups of principal neurons with repeated coincident firing within short time windows during the exposure. We opted for 25 ms windows because it was previously suggested that neuronal co-activity at this timescale is optimal for cell assembly expression (Harris et al., 2003). A two-step statistical method first estimated the number of significant co-activation patterns in the spike trains and then extracted those patterns with an independent component analysis (Figure S2A; Lopes-dos-Santos et al., 2013). A total of 521 patterns (5.6 ± 0.2 per block) were identified, each described by a weight vector containing the contribution of each neuron (Figures 1C and S2A). We confirmed that pairs of neurons with a large contribution to the same “assembly pattern” had far stronger instantaneous rate correlations than other neuron pairs (Figure 1D).

We next assessed whether the detected assembly patterns carried behaviorally relevant information. When we tracked the expression of each pattern over time (Figure S2B), we found that their activations were spatially tuned (Figure 1C). In line with this, the discharge of neurons with a large contribution to

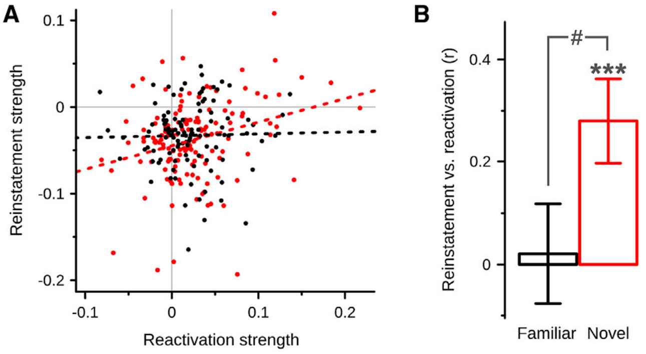

Figure 2. Assembly Pattern Reactivation Following a Novel, but Not Familiar, Enclosure Correlates with Upcoming Awake Reinstatement

(A) Scatterplot of the reinstatement strength (change in expression strength from exposure to re-exposure) versus the reactivation strength (change in expression strength from rest before to rest after; see also Figures S1F–S1H) of assembly patterns detected in the familiar (black) or novel (red) enclosure. Dashed lines are corresponding ordinary least-squares regression lines (familiar, slope = 0.02, p = 0.81, R^2 = 0.00; novel, slope = 0.27, p < 0.001, R^2 = 0.08).

(B) Correlation between reactivation and reinstatement strength is stronger after a novel enclosure than after the familiar one. Error bars represent ± 1 SE of the correlation coefficient; novel versus zero, ***p < 0.001; familiar versus novel, $^{\#}$p < 0.05.

the same pattern substantially overlapped in space (Figures 1C and 1E). Thus, although the assembly pattern detection was blind to the animal's location and solely based on short-timescale co-activations, it successfully grouped together neurons representing the same location. However, the detection was not merely governed by the spatial overlap of neurons' discharge, for distinct patterns of the same enclosure could overlap in space (e.g., the light green and orange patterns in Figure 1C). This suggests that a given location could be represented by several assembly patterns (Jackson and Redish, 2007).

We then applied this pattern detection method across the several recording blocks performed within each day to compare patterns expressed in distinct enclosures. We found that assembly patterns detected during exposure to a given enclosure were more similar to those detected during re-exposure to that same enclosure than to those detected in another enclosure on that day (Figure 1F; environment-specificity index, 0.18 ± 0.01; n = 237 patterns, p < 0.0001). Together, these results show that repeated short-timescale co-activations of hippocampal principal neurons form spatially selective assembly patterns that are reinstated upon context re-exposure.

Disruption of Reactivation Impairs Reinstatement of Assembly Patterns Representing Novel, but Not Familiar, Environments

We then checked whether the reinstatement of assembly patterns during context re-exposure correlated with their offline reactivation. To do so, the expression of assembly patterns identified during the exposure was tracked throughout each recording block (Figure 1B). We found that the average expression strength of 71.7% of these patterns was stronger during the rest following the exposure than during the rest preceding it (against chance, p < 0.0001; Figures S1F and S1G), confirming that waking assembly patterns were subsequently reactivated in the sleep box. Importantly, the reactivation strength of patterns expressed in novel enclosures, but not in the familiar one, correlated with their reinstatement strength during context re-exposure (Figure 2).

The reactivation of assembly patterns was strongest during SWRs (Figure S1H), in line with studies based on pairwise correlations (Wilson and McNaughton, 1994; O'Neill et al., 2008). Therefore, to test whether offline reactivation of assembly patterns is required for their subsequent awake reinstatement, we performed closed-loop optogenetic silencing of principal neurons during SWRs (Figure S3A). We injected the dorsal CA1 hippocampus of CamKII-Cre mice (n = 7) with a flex-ArchT-GFP viral construct to target principal neurons with the light-driven proton pump ArchT (Figure S3B). Mice were then implanted with tetrodes and optic fibers to monitor and manipulate neuronal discharge (Figure 1A). In rest sessions without light delivery, 80.1% ± 1.0% of the SWRs were detected in real time with an average latency of 7.68 ± 0.30 ms before their peak power. When light was delivered upon SWR detection, principal neurons were silenced within 3.07 ± 0.54 ms from the light onset and returned to baseline firing within 22.12 ± 1.01 ms following the light offset (Figures 3A, 3B, and S3C). We found that SWR silencing applied during rest following a novel enclosure impaired assembly pattern reinstatement during context re-exposure (Figures 3C, S3E, and S3F). Importantly, the same SWR silencing during rest following the familiar enclosure did not alter the reinstatement of its assembly patterns (Figure 3C; with interaction SWR-silencing x enclosure type, $F(1,318) = 5.05$, $p < 0.05$). Moreover, after random optogenetic silencing performed independently of SWR occurrence, patterns expressed in a novel enclosure were reinstated stronger than after SWR silencing (Figures S3C–S3E). These results, further confirmed using conventional neuron-pair and single-neuron analyses (Figures S3G and S3H), establish that offline reactivation during SWRs is required to stabilize newly expressed co-activation patterns.

Gradually Strengthened, but Not Early Stabilized, Assembly Patterns Require Reactivation

If repeated neuronal co-activation strengthens a newly formed assembly (Hebb, 1949), then the strength of the corresponding firing pattern would be expected to increase throughout its formation. To test for such a strengthening dynamic, we fitted a linear trend to the expression strength of each assembly pattern during the first exposure to a novel enclosure. We found a significant positive slope for 134 out of 335 patterns (40.0%), compared to only 18 patterns (5.4%) with a significant negative slope. We refer to the patterns with a significant increasing linear trend as "gradually strengthened" (Figure 4A). Interestingly, the remaining patterns showed a similar strengthening only during the first few minutes (Figure 4A). This initial positive trend could reflect the rapid recruitment of these patterns during the first exposure to an enclosure, and we refer to them as "early stabilized." Importantly, gradually strengthened and early stabilized patterns had similar composition of their weight vectors and were equally spatially selective (Figure 4B; Table S1).

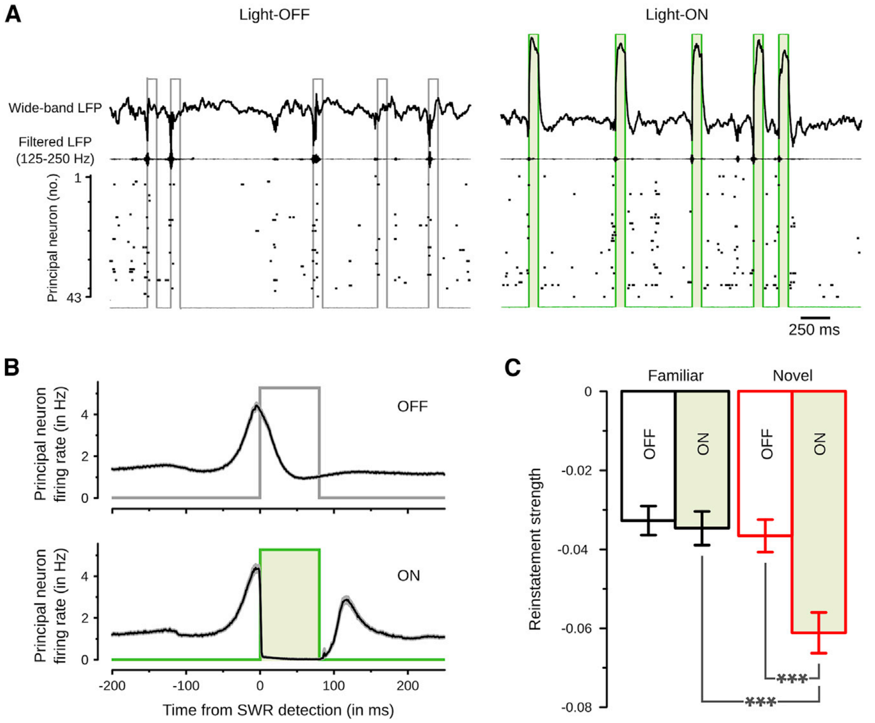

Figure 3. Optogenetic SWR Silencing Impairs Reinstatement of Assembly Patterns Associated with a Novel, but Not Familiar, Enclosure

(A and B) Closed-loop feedback transiently silencing principal neurons during SWRs is illustrated with a raw data example (A) and quantified by the firing rate response (mean ± SEM) of principal neurons (B; light-OFF, n = 1,988 neurons; light-ON, n = 1,527).

(C) After exposure to a novel enclosure, SWR silencing impairs the reinstatement of assembly patterns during context re-exposure (light-OFF, n = 139 patterns; light-ON, n = 136). This is not the case following exposure to the familiar enclosure (light-OFF, n = 108 patterns; light-ON, n = 78). As reinstatement strength is defined by the change in a pattern's average expression strength from exposure to re-exposure, a null score corresponds to "perfect" reinstatement while the more negative, the worse the reinstatement. Data are represented as mean ± SEM; ***p < 0.001.

See also Figure S3.

We finally tested whether these concurrently expressed assembly patterns equally required offline reactivation for their lasting expression. Both sets of patterns were reactivated; the reactivation of the gradually strengthened patterns was stronger (Table S1). Importantly, only the reactivation strength of the gradually strengthened patterns, and not of the early stabilized ones, correlated with their future reinstatement during context re-exposure (Figure 4C). Moreover, SWR silencing only impaired the reinstatement of the gradually strengthened patterns (Figure 4D; with interaction SWR-silencing x pattern type, $F(1,271) = 6.28$, $p < 0.05$). In the baseline condition (i.e., no optogenetic silencing), the reinstatement of both early stabilized and gradually strengthened patterns was context dependent (Figure 4D; "light-OFF" versus "other enclosure"). SWR silencing decreased the reinstatement of gradually strengthened patterns down to their non-specific strength level seen in a different enclosure, but it did not significantly affect the reinstatement of the early stabilized patterns (Figure 4D; "light-ON" versus "other enclosure").

DISCUSSION

Our study establishes that the context-dependent reinstatement of hippocampal co-firing patterns requires SWR reactivation following their initial expression during spatial exploration. The idea that the stabilization of newly formed cell assembly patterns involves their reactivation during resting behavior has been a long-standing hypothesis central to many theories of memory consolidation, although it has never been directly tested. Here, by combining ensemble recordings with an unsupervised statistical framework, we identified short-timescale co-activation patterns of CA1 principal neurons, which we showed to be spatially selective. We observed that for only a specific set of these patterns, those with continued strengthening throughout their initial expression, the reinstatement during context re-exposure was both correlated with their reactivation and suppressed by optogenetic SWR silencing. This study therefore provides direct evidence that the stabilization of recently formed, space-representing hippocampal cell assembly patterns depends on offline reactivation.

Time-Limited Role of SWR Reactivation in the Persistence of Neuronal Representations of Space Could Underlie Memory Consolidation

Previous studies showed that post-learning disruption of sleep SWRs by electrical stimulation of the ventral hippocampal commissure impaired spatial memory performance (Girardeau et al., 2009; Ego-Stengel and Wilson, 2010), thereby laying the foundation for an instrumental role of SWRs in memory. However, it was not possible in these studies to establish whether the observed impairment was caused by the disruption of the SWR-associated reactivation of waking firing patterns, or due to an unspecific effect of electrical stimulation coupled to SWRs. Moreover, it remained to be tested whether the effect of SWR disruption depends on such a manipulation being applied shortly after encoding. Indeed, to decisively demonstrate that a process has a role in consolidation, it is required to show that its disruption has a time-limited effect, namely that its disruption affects the persistence of traces of recent experiences and not those of remote ones (Dudai, 2004; Squire et al., 2015).

Here, we directly silenced SWR reactivation using an optogenetic approach. We found that this intervention disrupted the upcoming reinstatement of hippocampal assembly patterns when performed after the first exploration of a (thus novel) environment. Importantly, the same SWR silencing was ineffective on

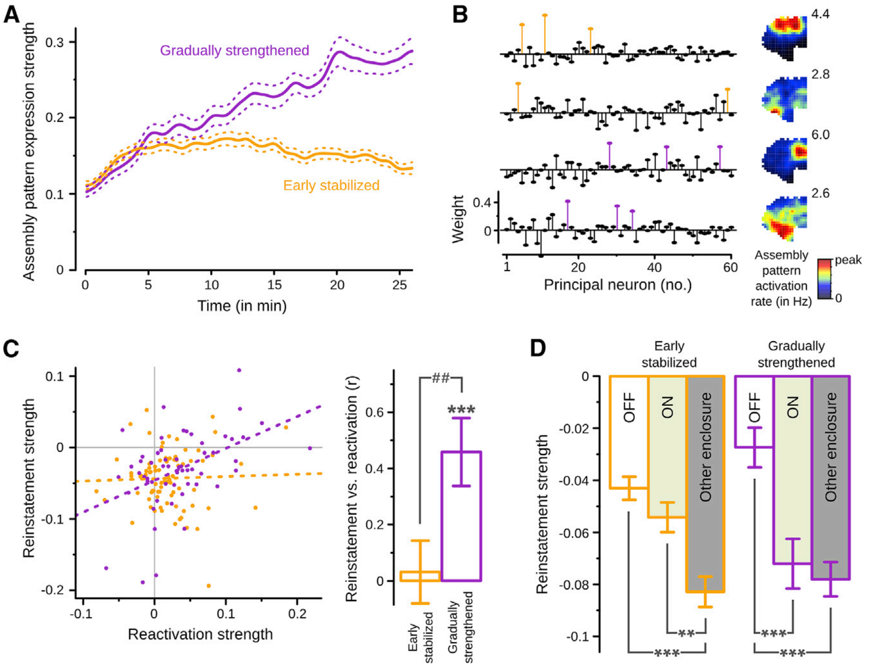

Figure 4. Offline Reactivation Is Required for Reinstatement of Gradually Strengthened, but Not of Early Stabilized, Assembly Patterns

(A) The expression strength (mean ± SEM) of gradually strengthened patterns (purple; n = 134) continually increases during the exposure session, while that of early stabilized patterns (orange; n = 201) is more stable. Yet both sets are equally strengthened in the first few minutes.

(B) Examples of two early stabilized (top) assembly patterns simultaneously expressed with two gradually strengthened (bottom) assembly patterns.

(C) In the light-OFF condition, the reactivation of gradually strengthened patterns, but not of early stabilized ones, correlates with their reinstatement strength during context re-exposure. Dashed lines are corresponding ordinary least-squares regression lines (early stabilized, slope = 0.03, p = 0.78, R^2 = 0.00; gradually strengthened, slope = 0.45, $p < 0.001$, R^2 = 0.21). Error bars represent ± 1 SE of the correlation coefficient; gradually strengthened versus zero, ***$p < 0.001$; early stabilized versus gradually strengthened, ##$p < 0.01$.

(D) SWR silencing does not impair the context-dependent reinstatement of early stabilized patterns (light-OFF, n = 82 patterns; light-ON, n = 83; other enclosure, n = 155), but causes the reinstatement of gradually strengthened patterns to drop to the unspecific level at which they are expressed in a distinct enclosure of another recording block that day (light-OFF, n = 57 patterns; light-ON, n = 53; other enclosure, n = 103). Data are represented as mean ± SEM; **$p < 0.01$, ***$p < 0.001$.

See also Table S1.

pattern reinstatement when performed after an environment that had been repetitively experienced before (hence familiar). This control condition rules out a generic effect of SWR disruption. Indeed, the familiar environment is here a "delayed-block condition" that establishes the time-limited role of SWR reactivation. Our results, combined with the previously demonstrated behavioral effects, provide converging evidence that SWR reactivation supports memory consolidation by stabilizing the underlying cell assemblies.

The lack of effect of SWR silencing on the reinstatement of "familiar" assembly patterns raises the question of why these patterns are still reactivated. One explanation could be that the repetitive explorations of a given environment lead to the formation of multiple "entry points" to the same assemblies. This is reminiscent of the idea that re-experiencing a given memory is associated with the formation of multiple neuronal traces (Moscovitch et al., 2006). In this scenario, reactivation following exploration of the familiar environment might still stabilize some of these additional traces, but SWR silencing is ineffective because previously stabilized traces are sufficient to retrieve the assembly patterns representing that environment. Another possibility is that reactivation no longer stabilizes "familiar" patterns within the hippocampus, but still contributes to their "transfer" to downstream circuits (Squire et al., 2015).

Early Stabilized versus Gradually Strengthened Assembly Patterns

Our study shows that for those assembly patterns that had an increasing expression strength over continued experience in a novel environment, their subsequent reinstatement was correlated with their reactivation and disrupted by SWR silencing. The features of this set of patterns are consistent with the Hebbian postulate of "fire together, wire together." Conversely, the reinstatement of the other, concurrently expressed patterns that were no longer strengthened after the first few minutes of exploration was not correlated with their reactivation and unaffected by SWR silencing. Yet both sets were equally spatially selective and thus appeared to carry a similar representational attribute. These findings indicate that concurrent space-representing assembly patterns can markedly differ in their plastic properties.

The early stabilized patterns might have gained independence from offline reactivation because they rapidly acquired the status of "familiar" patterns while stably expressed during the exposure session. In this scenario, their consolidation would take place "on line" and the inefficacy of SWR silencing on these patterns would be an extreme reflection of the time-limited role of SWR reactivation. Perhaps, early stabilized patterns could be quickly consolidated because the place fields of their contributing neurons remap more coherently in the novel enclosure, for example, according to a topographical transform, rather than unpredictably (cf. Figure S1E). Another, non-exclusive possibility is that the early stabilized patterns are more "hardwired" to represent the new spatial layout due to the specifics of their contributing neurons in terms of existing spatial inputs or intrinsic properties. The gradually strengthened patterns, in contrast, could gain their spatial selectivity and increased strength by more plastic changes throughout the exploration. Under this scenario, the

difference in strength between both sets of patterns could reflect that such plasticity enables neurons to better synchronize with their peers. An interesting related hypothesis is that the early stabilized patterns could provide a "nearly automatic" and yet stable representation of space, "ready to use" by downstream circuits, for instance, for (immediate) navigational purposes. As the animal accumulates experiences in the environment, the strengthening of the gradually strengthened patterns could reflect the formation of additional, perhaps richer, memory traces (Buzsáki, 2015b; Schiller et al., 2015).

Conclusions

Altogether, this study establishes that the lasting expression of recently formed hippocampal co-activation patterns that resemble classical Hebbian assemblies requires their offline reactivation. Our findings support the long-standing hypothesis of an instrumental role of offline SWR reactivation in the consolidation of memory-representing assemblies. However, reactivation-dependent assembly patterns were co-expressed with other space-coding patterns that did not require offline reactivation. As a pattern's dependency on offline SWR reactivation was related to its strengthening dynamics during spatial encoding, this study therefore highlights functional heterogeneity within co-expressed representations of space.

EXPERIMENTAL PROCEDURES

Full details of the procedures are provided in the Supplemental Experimental Procedures.

Animals, Ensemble Recordings, and Optogenetic Silencing

All animals used were male adult transgenic CamKIIa-Cre mice (RRID: IMSR_JAX:005359). To silence principal neurons, mice were injected with a Cre-dependent ArchT-GFP viral vector into the dorsal CA1 hippocampus. CamKII::ArchT mice were then implanted with ten tetrodes combined with two optic fibers to monitor and manipulate the activity of CA1 principal neurons (Figures 1A and S4). Each mouse performed multiple recording blocks (Figure 1B) per day. Every day, the animal was first recorded in its sleep box ("rest before"; ~25 min). For each recording block, the animal was then allowed to successively explore an open-field enclosure ("exposure"; ~25 min), rest for 1 hr in its sleep box ("rest after"), and again explore the same enclosure ("re-exposure"; ~25 min). The open field was either familiar (i.e., repeatedly explored prior to the recordings) or novel (i.e., never seen before). In some recording blocks, optogenetic SWR silencing was performed during the "rest after." For this, SWRs were detected in real time using the ripple-frequency band power to trigger delivery of a 561 nm light pulse (Figure S3A). In some other blocks, random silencing was instead performed, with a matched number of light pulses delivered independently of SWRs. All experiments involving animals were conducted according to the UK Animals (Scientific Procedures) Act 1986 under personal and project licenses issued by the Home Office following ethical review.

Assembly Pattern Analysis

Neuronal co-firing patterns were detected using an unsupervised statistical framework based on independent component analysis. Spikes of each principal neuron were counted in 25 ms time bins covering the exposure session. To avoid a bias toward neurons with higher firing rates, the binned spike counts were z scored. Assembly patterns were then extracted in a two-step procedure (Figure S2A). First, the number of significant co-activation patterns embedded within the dataset was estimated as the number of principal component variances above a threshold derived from an analytical probability function for uncorrelated data (Marčenko-Pastur distribution). Then, an independent component analysis extracted the assembly patterns from the projection of the data into the subspace spanned by these significant principal components.

To track the expression of these assembly patterns over time (Figure S2B), a projection matrix was constructed for each pattern from the outer product of its weight vector. This allowed the computation of the similarity between each pattern and the recorded firing activity at any given time. The main diagonal of the projection matrix was set to zero to ensure that only co-activations of at least two neurons could contribute to the expression of a pattern. To achieve a high temporal resolution, the spike train of each neuron was convolved with a Gaussian kernel (and then z scored). The expression strength of a pattern at any point in time was then defined as the quadratic form of its projection matrix with the smoothed and z scored firing rate vector. The assembly pattern activations used to compute assembly maps were defined as peaks in the expression strength above 5. Note that each detected pattern had many of such activations over time (average activation rate = 0.95 ± 0.02 Hz). For each pattern detected during the exposure, its reactivation strength was defined as the difference in its average expression strength during "rest after" minus that during "rest before." Its reinstatement strength was similarly defined as the difference in its average expression strength during "re-exposure" minus that during the "exposure." Patterns detected in a novel enclosure were classified as gradually strengthened if a significant positive linear trend could be fitted to their expression strength during the exposure, and as early stabilized otherwise.

Statistical Analysis

Details of all performed statistical tests are provided in the Supplemental Experimental Procedures. All tests based on a test statistic with a symmetric distribution were performed two sided. Reported group data are mean ± SEM, unless stated otherwise.

SUPPLEMENTAL INFORMATION

Supplemental Information includes Supplemental Experimental Procedures, four figures, and one table and can be found with this article online at http://dx.doi.org/10.1016/j.neuron.2016.10.020.

A video abstract is available at http://dx.doi.org/10.1016/j.neuron.2016.10.020#mmc3.

AUTHOR CONTRIBUTIONS

Conceptualization, G.M.v.d.V. and D.D.; Methodology, G.M.v.d.V. and D.D.; Software, G.M.v.d.V., C.G.M., K.A., and D.D.; Formal Analysis, G.M.v.d.V.; Investigation, G.M.v.d.V. and S.T.; Writing – Original Draft, G.M.v.d.V. and D.D.; Writing – Reviewing & Editing, G.M.v.d.V., S.T., C.G.M., K.A., and D.D.; Visualization, G.M.v.d.V.; Supervision, D.D.; Funding Acquisition, D.D.

ACKNOWLEDGMENTS

We thank T. Klausberger, A. Peyrache, and V. Lopes-dos-Santos for their comments on a previous version of the manuscript; N. Campo-Urriza and J. Janson for technical assistance; N. Campo-Urriza, B. Micklem, and L. Katona for visualization of tetrode tracks; and E.S. Boyden (MIT) for sharing the flex-ArchT-GFP construct. K.A. is funded by the Deutsche Forschungsgemeinschaft (SFB 1134). This work was supported by the Medical Research Council UK (awards MC_UU_12020/7, MC_UU_12024/3, MC_ST_U13067, and MC_ST_U11056).

Received: March 25, 2016
Revised: August 16, 2016
Accepted: September 21, 2016
Published: November 10, 2016

REFERENCES

Barnes, C.A., Suster, M.S., Shen, J., and McNaughton, B.L. (1997). Multistability of cognitive maps in the hippocampus of old rats. Nature *388*, 272–275.

CellPress

Buzsáki, G. (2010). Neural syntax: cell assemblies, synapsembles, and readers. Neuron *68*, 362–385.

Buzsáki, G. (2015a). Hippocampal sharp wave-ripple: a cognitive biomarker for episodic memory and planning. Hippocampus *25*, 1073–1188.

Buzsáki, G. (2015b). Neuroscience. Our skewed sense of space. Science *347*, 612–613.

Dudai, Y. (2004). The neurobiology of consolidations, or, how stable is the engram? Annu. Rev. Psychol. *55*, 51–86.

Ego-Stengel, V., and Wilson, M.A. (2010). Disruption of ripple-associated hippocampal activity during rest impairs spatial learning in the rat. Hippocampus *20*, 1–10.

Girardeau, G., Benchenane, K., Wiener, S.I., Buzsáki, G., and Zugaro, M.B. (2009). Selective suppression of hippocampal ripples impairs spatial memory. Nat. Neurosci. *12*, 1222–1223.

Harris, K.D., Csicsvari, J., Hirase, H., Dragoi, G., and Buzsáki, G. (2003). Organization of cell assemblies in the hippocampus. Nature *424*, 552–556.

Hebb, D.O. (1949). The Organization of Behavior: A Neuropsychological Theory (Wiley).

Jackson, J., and Redish, A.D. (2007). Network dynamics of hippocampal cell-assemblies resemble multiple spatial maps within single tasks. Hippocampus *17*, 1209–1229.

Kentros, C.G., Agnihotri, N.T., Streater, S., Hawkins, R.D., and Kandel, E.R. (2004). Increased attention to spatial context increases both place field stability and spatial memory. Neuron *42*, 283–295.

Leutgeb, S., Leutgeb, J.K., Barnes, C.A., Moser, E.I., McNaughton, B.L., and Moser, M.-B. (2005). Independent codes for spatial and episodic memory in hippocampal neuronal ensembles. Science *309*, 619–623.

Lopes-dos-Santos, V., Ribeiro, S., and Tort, A.B.L. (2013). Detecting cell assemblies in large neuronal populations. J. Neurosci. Methods *220*, 149–166.

McNamara, C.G., Tejero-Cantero, Á., Trouche, S., Campo-Urriza, N., and Dupret, D. (2014). Dopaminergic neurons promote hippocampal reactivation and spatial memory persistence. Nat. Neurosci. *17*, 1658–1660.

Moscovitch, M., Nadel, L., Winocur, G., Gilboa, A., and Rosenbaum, R.S. (2006). The cognitive neuroscience of remote episodic, semantic and spatial memory. Curr. Opin. Neurobiol. *16*, 179–190.

O'Keefe, J., and Dostrovsky, J. (1971). The hippocampus as a spatial map. Preliminary evidence from unit activity in the freely-moving rat. Brain Res. *34*, 171–175.

O'Neill, J., Senior, T.J., Allen, K., Huxter, J.R., and Csicsvari, J. (2008). Reactivation of experience-dependent cell assembly patterns in the hippocampus. Nat. Neurosci. *11*, 209–215.

O'Neill, J., Pleydell-Bouverie, B., Dupret, D., and Csicsvari, J. (2010). Play it again: reactivation of waking experience and memory. Trends Neurosci. *33*, 220–229.

Rasch, B., and Born, J. (2007). Maintaining memories by reactivation. Curr. Opin. Neurobiol. *17*, 698–703.

Sadowski, J.H.L.P., Jones, M.W., and Mellor, J.R. (2016). Sharp-wave ripples orchestrate the induction of synaptic plasticity during reactivation of place cell firing patterns in the hippocampus. Cell Rep. *14*, 1916–1929.

Schiller, D., Eichenbaum, H., Buffalo, E.A., Davachi, L., Foster, D.J., Leutgeb, S., and Ranganath, C. (2015). Memory and space: towards an understanding of the cognitive map. J. Neurosci. *35*, 13904–13911.

Singer, A.C., and Frank, L.M. (2009). Rewarded outcomes enhance reactivation of experience in the hippocampus. Neuron *64*, 910–921.

Squire, L.R., Genzel, L., Wixted, J.T., and Morris, R.G. (2015). Memory consolidation. Cold Spring Harb. Perspect. Biol. *7*, a021766.

Wilson, M.A., and McNaughton, B.L. (1993). Dynamics of the hippocampal ensemble code for space. Science *261*, 1055–1058.

Wilson, M.A., and McNaughton, B.L. (1994). Reactivation of hippocampal ensemble memories during sleep. Science *265*, 676–679.

Neuron, Volume *92*

Supplemental Information

Hippocampal Offline Reactivation Consolidates Recently Formed Cell Assembly Patterns during Sharp Wave-Ripples

Gido M. van de Ven, Stéphanie Trouche, Colin G. McNamara, Kevin Allen, and David Dupret

SUPPLEMENTAL FIGURES and TABLE

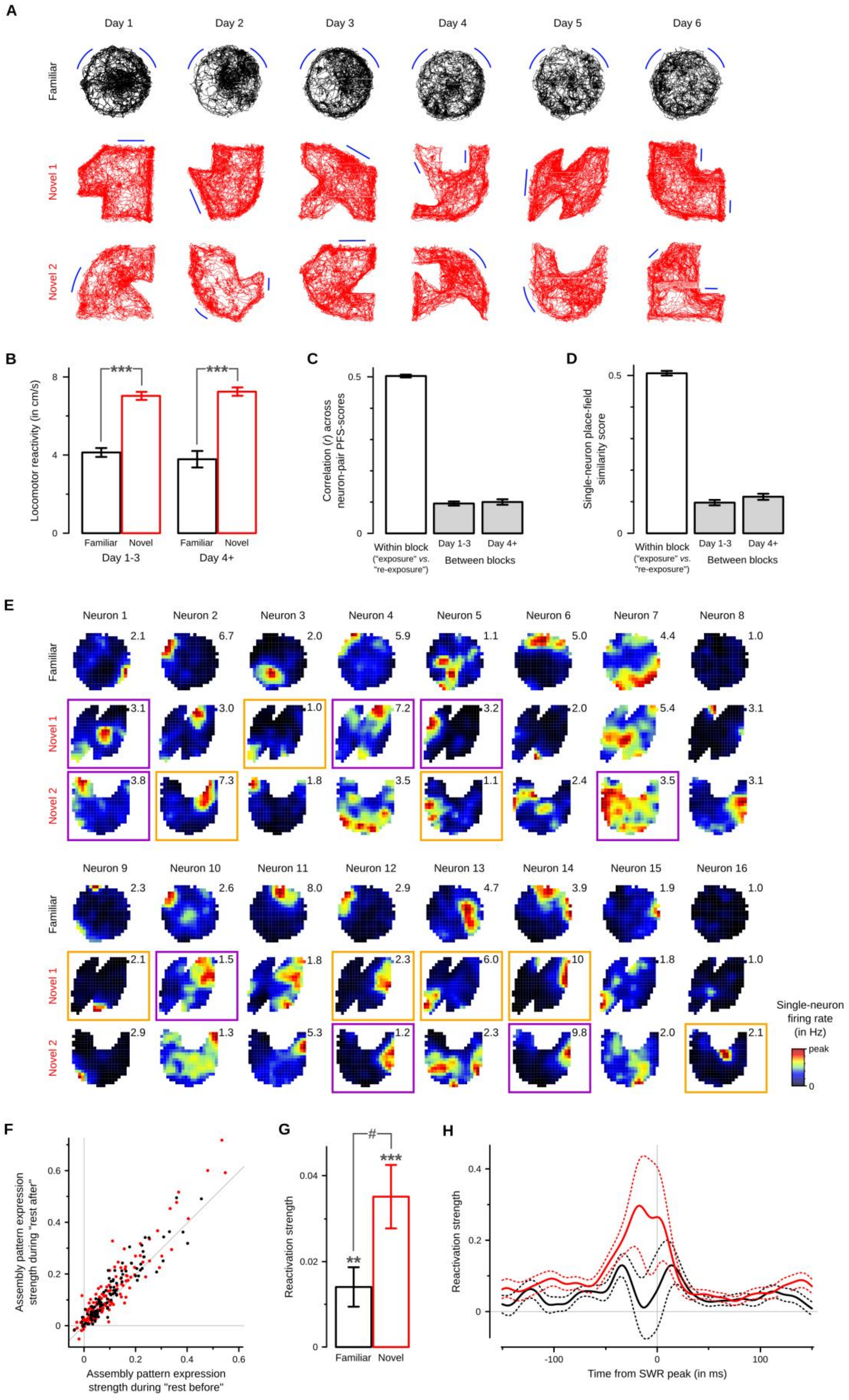

Figure S1. *(Refers to Figures 1 and 2)* Comparisons between “familiar” and “novel” recording blocks

(A) Example paths from one mouse recorded during various exposure sessions. Each path depicted corresponds to the open-field enclosure of one recording block. Besides differences in geometrical shape, the enclosures differed in the cue-cards (here schematically illustrated by blue lines) lining the walls. Note the variety of enclosures a given animal explored throughout the entire recording experiment. The particular order in which the “familiar” and “novel” recording blocks were presented varied between recording days.
(B) Locomotor reactivity to open-field enclosure, as quantified by the average locomotion speed during the first 5 min of the exposure session. Note that the locomotion speed is substantially higher in a novel than in the familiar enclosure, which indicates that mice successfully detected spatial novelty (e.g., Berke et al., 2008). This difference in speed is consistently observed during the first recording days (day 1-3, familiar: n = 19 sessions; day 1-3, novel: n = 28) and the last ones (day 4+, familiar: n = 11; day 4+, novel: n = 35), indicating that also towards the end of the recording experiment the “novel” enclosures explored by these mice are indeed treated as novel. Data are represented as mean ± SEM; *** P < 0.001, unpaired two-sample *t*-test.
(C-D) Quantification of principal neuron remapping across enclosures. (C) Pearson correlation coefficient of the neuron-pair place-field similarity (PFS; see **Supplemental Experimental Procedures**) scores between the indicated sessions, calculated over all principal neuron pairs (within recording block: n = 35,764 neuron-pairs; between blocks, day 1-3: n = 22,877; between blocks, day 4+: n = 13,004). Error bars represent ± SE of the correlation coefficient. (D) Single-neuron place field similarity score (see **Supplemental Experimental Procedures**) between the indicated sessions, averaged over all recorded principal neurons (mean ± SEM; within block: n = 1792 neurons; between blocks, day 1-3: n = 1207; between blocks, day 4+: n = 969). Note that the similarity score is calculated based on only those spatial bins that were visited by the animal during both sessions considered. Both measures show that the firing activity of principal neurons remaps between different enclosures, compared to repeated exposures to the same enclosure. This hippocampal remapping is consistently observed during both the first (day 1-3) and the last (day 4+) recording days, which provides further evidence that mice distinguished between different enclosures throughout the entire recording experiment.
(E) Example single-neuron firing rate maps expressed during the exposure sessions by a set of CA1 principal neurons simultaneously recorded throughout three recording blocks. The top right number of each map is the peak firing rate (in Hz). Warm colors (red) correspond to high firing rate regions (i.e., the place field) of the neuron in that enclosure. For both novel enclosures explored on that day by this mouse, color coded squares indicate neurons with high contribution to an early stabilized (orange) and/or to a gradually strengthened (purple) assembly pattern detected in that enclosure (see also the remapping section of **Table S1**).
(F) The average expression strength of most assembly patterns identified during the exposure session is stronger during the sleep/rest session after this exposure than during the sleep/rest before. This indicates the presence of assembly pattern reactivation. Note that two data-points (Novel: [0.85, 0.89] and [0.53, 1.15], both above the diagonal) are outside this graph’s axes.
(G) The reactivation strength (expression strength during “sleep/rest after” minus expression strength during “sleep/rest before”) of assembly patterns is substantially stronger in the first 20 min after exposure to a novel enclosure (n = 139 assembly patterns) than in the first 20 min after exposure to the familiar enclosure (n = 108). Note that also after exposure to the familiar enclosure the average reactivation strength is significantly above zero, which indicates again that assembly patterns are also reactivated after familiar environments. Data are represented as mean ± SEM; ** P < 0.01, *** P < 0.001, one-sample *t*-test versus zero; $^{\#}$ P < 0.05, unpaired two-sample t-test.
(H) Assembly pattern reactivation is emphasised during sharp wave-ripple (SWR) events, particularly after exposure to a novel enclosure. Solid lines represent mean difference in expression strength between sleep/rest after and sleep/rest before, dashed lines ±1 SEM (black: familiar, red: novel). Both the mean- and SEM-traces are smoothed with a Gaussian kernel with SD = 4 ms.

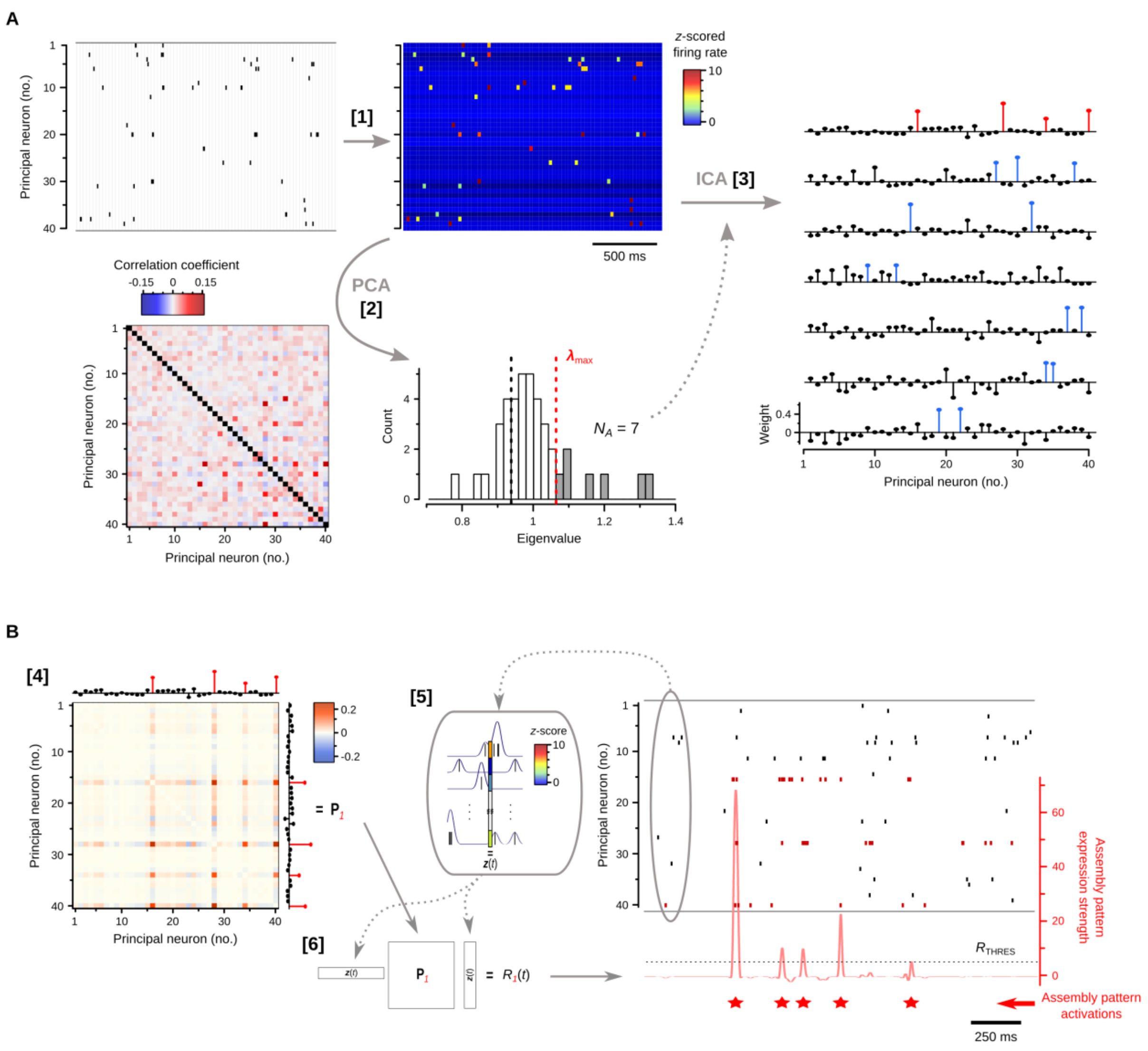

Figure S2. *(Refers to Figure 1 and Experimental Procedures)* Identification and tracking of assembly patterns

(A) Assembly pattern identification. The different steps to identify neuronal co-activation patterns are illustrated for 40 simultaneously recorded principal neurons. After binning (25 ms time bins) and normalizing (*z*-score) each neuron's spike counts **[1]**, principal component analysis (PCA) is applied to the resulting correlation matrix of the binned (and *z*-scored) spike count matrix to find the number of patterns describing statistically significant co-activations between neurons **[2]**, as estimated by the number of eigenvalues N_A exceeding the Marcenko-Pastur threshold $\lambda_{\max}$. Independent component analysis (ICA) is then used to identify N_A assembly patterns, given by weight vectors indicating the contribution of each neuron to that pattern **[3]**. For each pattern, neurons with a high contribution (i.e., weight exceeding two standard deviations above the mean) are colored for display purpose (red for the first assembly pattern, blue for all the other patterns). For clarity, only a few second sample of the spike trains raster plot and the z-scored spike count matrix are shown; the assembly patterns are however identified using the time bins spanning each entire exposure session.
(B) Tracking of assembly pattern expression strength. For each assembly pattern, a projector matrix is constructed by taking the outer product matrix of its weight vector and setting the diagonal to zero (to ensure only co-activations of neurons can contribute to the expression of an assembly pattern) **[4]**. The spike trains are convolved with a Gaussian kernel **[5]**. The assembly pattern expression strength is then taken as the quadratic form of the projector matrix with the convolved and *z*-scored spike trains **[6]**. The scale of the expression strength can thus be interpreted as projected *z*-scores onto the weight vector of the assembly pattern. Assembly pattern activations are defined as peaks in the expression strength exceeding $R_{\text{THRES}} = 5$.

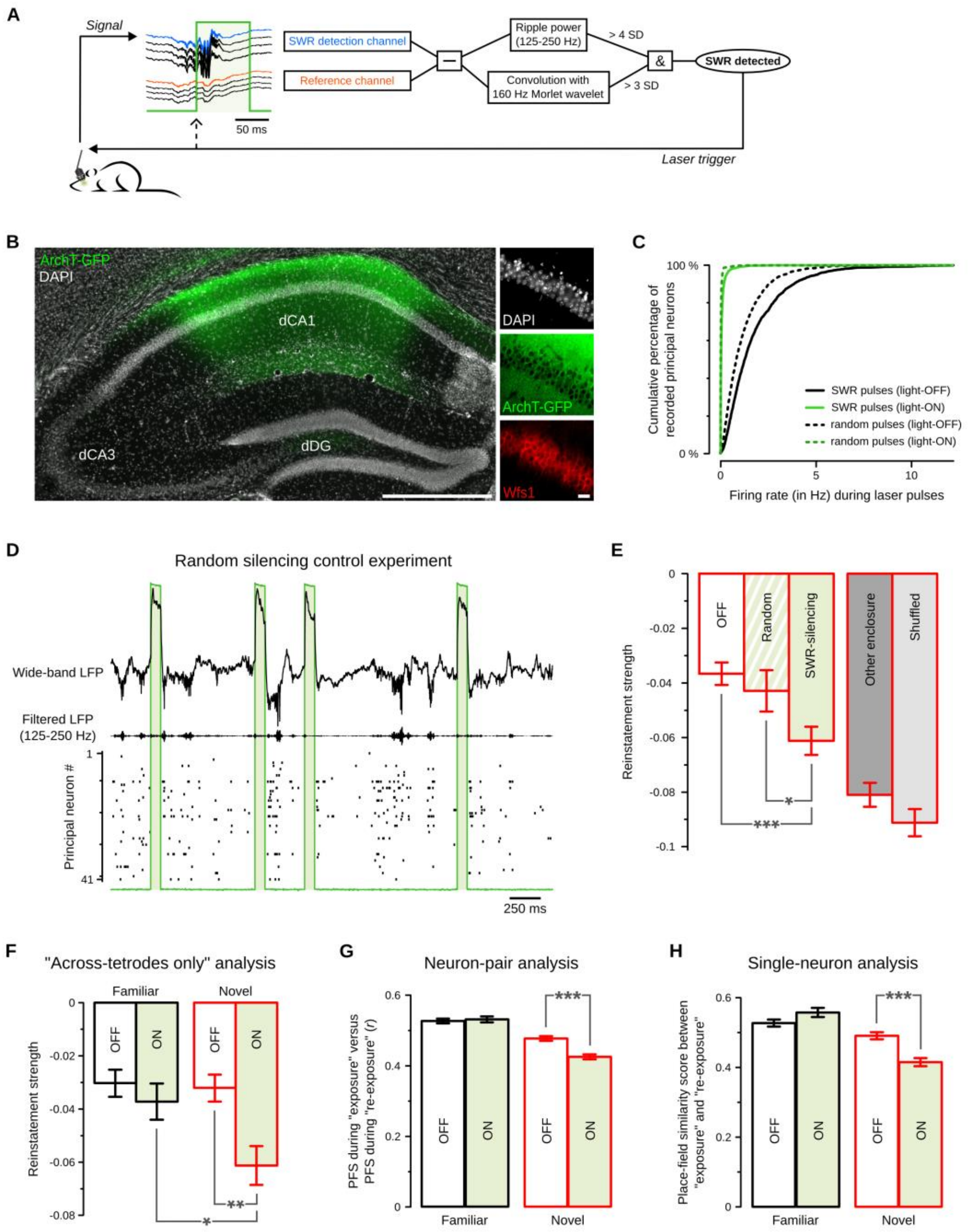

Figure S3. *(Refers to Figure 3)* Optogenetic silencing of dorsal CA1 principal neurons triggered by on line detected SWRs impairs upcoming reinstatement of hippocampal spatial maps representing novel environments

(A) Schematic of the on line SWR detection method (see **Supplemental Experimental Procedures**).
(B) Representative image showing ArchT-GFP expression restricted to the CA1 region of the dorsal hippocampus (dCA1) from a CamKII-Cre mouse (left; scale bar: 500 µm; dCA3 = dorsal CA3; dDG = dorsal Dentate Gyrus). The high-magnification images (right; scale bar: 20 µm) show the dCA1 pyramidal cell layer marked by dapi labelling of cell nuclei (top) and with expression of both the GFP reporter (middle) and Wolfram syndrome 1 (Wfs1; bottom), a specific marker for CA1 principal neurons. Quantification of Wfs1-immunopositive cells expressing ArchT-GFP in dCA1-injected CamKII::ArchT mice revealed high-level viral transfection of dCA1 principal neurons (90.8 ± 2.5 %, *n* = 8 sections from 2 mice).
(C) Cumulative frequency histogram of principal neuron firing rate calculated during SWR triggered pulses without the laser powered (*n* = 1988 neurons), during SWR triggered pulses with the laser powered (*n* = 1639), during randomly triggered pulses without the laser powered (*n* = 1988) and during randomly triggered pulses

with the laser powered (random silencing; *n* = 459). Note that 94.7 % of principal neurons fire less than 0.25 Hz during laser-powered pulses, thus showing the high-level of efficacy of ArchT silencing.
(D) Raw data example from a random optogenetic silencing session. *Top trace*, wideband local field potential (LFP). *Bottom trace*, 125–250 Hz band pass filtered signal highlighting ripple frequency events. *Raster plot*, spike times from simultaneously recorded dCA1 principal neurons (one neuron per row). Note the firing synchrony of principal neurons during SWR events that is spared by random optogenetic silencing performed independently of SWR occurrence.
(E) After exposure to a novel enclosure, SWR silencing (*n* = 136 assembly patterns, same data as in **Figure 3C**) impairs the upcoming reinstatement of assembly patterns during context re-exposure compared to when optogenetic silencing is not applied (light-OFF; *n* = 139, same data as in **Figure 3C**), but also compared to when principal neurons are randomly silenced (random; *n* = 60 assembly patterns from 5 mice). Note that because the reinstatement strength is defined as the change in a pattern's average expression-strength from exposure to re-exposure, a null score would correspond to a "perfect" reinstatement while the more negative the worse the reinstatement. Further note that even in the baseline light-OFF condition, the average reinstatement strength is negative, which reflects that recalled "memory" patterns are not exact copies of those initially formed. For comparison purposes, also displayed are the reinstatement strength obtained in another enclosure (*n* = 258 assembly patterns) and the reinstatement strength obtained after shuffling the spike-identities during the re-exposure session (*n* = 335). The latter is displayed to show the expression strength level that could be expected with random coincidence of neuronal spike discharge. For the shuffling, all spikes discharged by principal neurons were randomly re-assigned to the pool of recorded principal neurons, under the restriction that for each neuron the total number of spikes assigned to it remained the same. Note that this shuffling-procedure thus preserves the average firing rate of each neuron and also preserves, for any given time-window, the "population rate". This shuffling-procedure is the same as the "SWAP"-method used in Jackson et al., 2006. Data are represented as mean ± SEM; * P < 0.05, *** P < 0.001, unpaired two-sample *t*-test.
(F) In order to restrict the central analysis of this study, namely the effect of SWR silencing on the reinstatement of "familiar" and "novel" assembly patterns, to neurons recorded exclusively from different tetrodes, here we only considered assembly patterns with for each tetrode at most one neuron with high contribution (> 2 s.d. above the mean) to that pattern. With such a conservative analysis we also find that SWR silencing impairs the reinstatement of "novel" assembly patterns (light-OFF: *n* = 54 assembly patterns; light-ON: *n* = 39), but not that of the "familiar" patterns (light-OFF: *n* = 82; light-ON: *n* = 75). This result is thus consistent with that shown for all assembly patterns in **Figure 3C**. Data are represented as mean ± SEM; * P < 0.05, ** P < 0.01, unpaired two-sample *t*-test.
(G-H) The differential effect of SWR silencing on the reinstatement of neuronal representations of familiar versus novel space, is confirmed using conventional neuron-pair and single-neuron analyses. (G) A pairwise spatial map similarity measure (e.g., see McNamara et al., 2014), which is calculated over all across-tetrodes principal neuron pairs as the Pearson correlation coefficient between their place-field similarity (PFS; see **Supplemental Experimental Procedures**) scores during the exposure session and during the subsequent re-exposure (familiar, light-OFF: *n* = 17,256 neuron-pairs; familiar, light-ON: *n* = 10,018; novel, light-OFF: *n* = 18,508; novel, light-ON: *n* = 16,335). Error bars represent ± SE of the correlation coefficient; *** P < 0.001, *z*-test whether two correlations are different. (H) A single-neuron place field similarity score (e.g., see Kentros et al., 1998) is calculated for each principal neuron as the Pearson correlation between its spatial firing rate maps during the exposure session and during the subsequent re-exposure (familiar, light-OFF: *n* = 788 neurons; familiar, light-ON: *n* = 519; novel, light-OFF: *n* = 1004; novel, light-ON: *n* = 927;). Data are represented as mean ± SEM; *** P < 0.001, unpaired two-sample *t*-test.

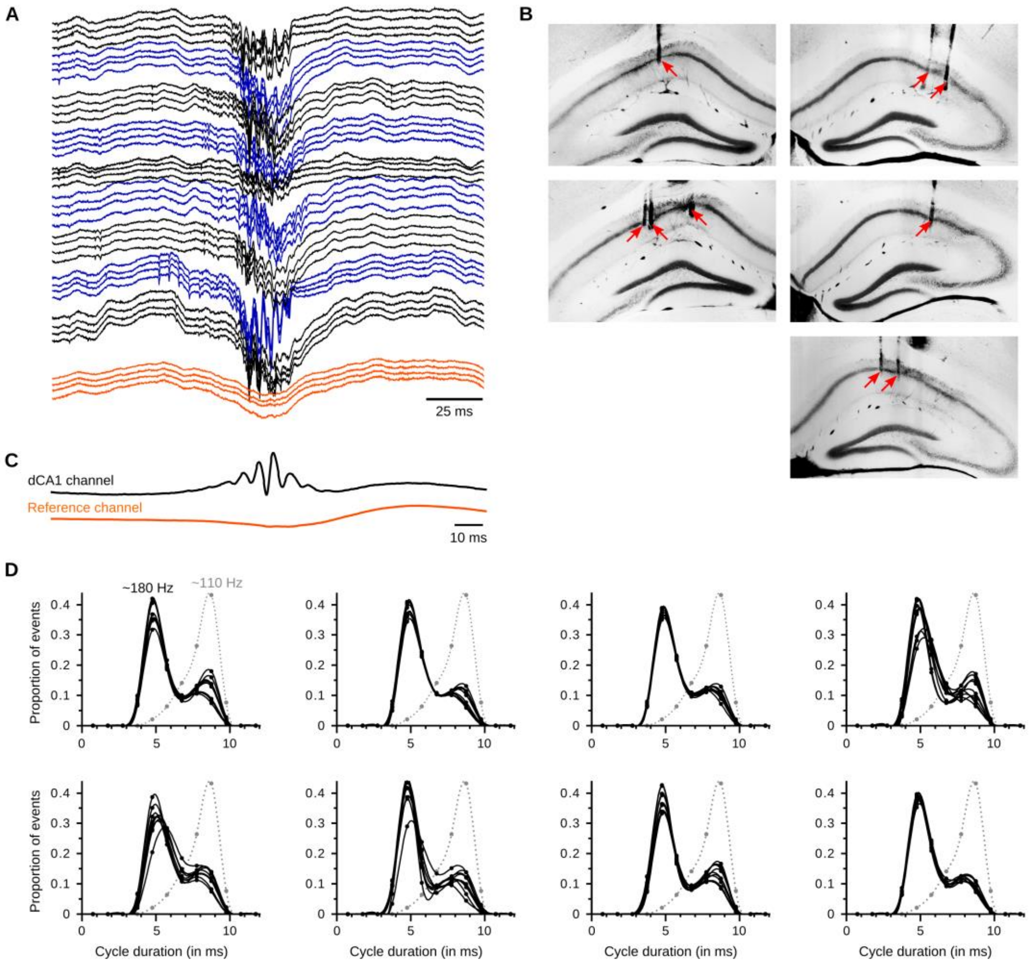

Figure S4. *(Refers to Experimental Procedures)* Recordings were performed from the CA1 region of the dorsal hippocampus as shown from the tetrode tracks and the SWR electrophysiological profile

(A) Example wideband local field potential signals from all tetrodes of one of the recorded mice. Nine of these tetrodes were daily lowered to the pyramidal cell layer of the CA1 region of the dorsal hippocampus (dCA1), as indicated by the presence of SWR events. For clarity, the signal from all channels of the same dCA1 tetrode (4 wire-channels per tetrode) is color coded in black or dark blue. The tenth tetrode (orange) was left above the hippocampus and used as a reference for SWR detection (see **Figure S3A**).
(B) Pictures of the osmium-stained coronal brain sections showing the tracks from the nine dCA1 tetrodes used to record the raw traces shown in (A). Red arrows indicate the tip of the tetrodes.
(C) Average SWR triggered waveforms from a channel of a dCA1 tetrode and of the reference tetrode. Note the presence of ripples (~5-6 ms long cycles) in the dCA1, but not the reference, tetrode.
(D) Distribution of the cycle duration of the fast oscillatory events detected from the hippocampal tetrodes of each recorded mice (one panel per mouse; each tetrode from which principal neurons were recorded is represented by one black curve; top-left panel is of the same mouse as in (A-C)). The oscillatory frequency (in Hz) of the detected events can be calculated by $1000/x$, with x the cycle duration (in ms). For visualisation purposes, in each panel the consecutive data points of each tetrode are connected via a cubic spline curve after rendering the data monotonic. Note the bimodal distribution of the oscillatory events detected from all recorded hippocampal tetrodes (black data points and connecting curves) with ~180 Hz and ~110 Hz frequency peaks, which is consistent with their dCA1 location (Csicsvari et al., 1999, 2000). For comparison, the cycle duration distribution of the fast oscillatory events detected from a tetrode located in the CA3 region of the dorsal hippocampus of another mouse (not included in this study’s analyses; gray data points and dotted curve) is shown on each panel; note in this case the unimodal distribution of the oscillatory events with a ~110 Hz frequency peak, consistent with previous studies (Csicsvari et al., 1999, 2000).

Variable	Early stabilized	Gradually strengthened	P-value
Pattern composition			
Sparsity of weight vector	0.64 ± 0.01	0.63 ± 0.01	P = 0.44
Neurons with high contribution (%)	5.03 ± 0.14	5.27 ± 0.19	P = 0.29
Expression strength			
First quarter of exposure session	0.148 ± 0.006	0.141 ± 0.009	P = 0.56
Last quarter of exposure session	0.147 ± 0.006	0.279 ± 0.014	P < 0.0001
Offline reactivation			
Reactivation strength	0.019 ± 0.005	0.045 ± 0.013	P < 0.05
Spatial selectivity			
Assembly map coherence	0.85 ± 0.02	0.89 ± 0.03	P = 0.25
Assembly map sparsity	0.56 ± 0.01	0.57 ± 0.02	P = 0.55
Assembly field size (bins)	37.1 ± 1.9	37.5 ± 2.3	P = 0.89
Environment-specificity index	0.14 ± 0.02	0.17 ± 0.02	P = 0.34
Spatial remapping of neurons with high contribution			
Single-neuron place field similarity score	0.16 ± 0.02	0.14 ± 0.03	P = 0.62
Correlation of member-pairs' PFS-scores	0.12 ± 0.07	0.15 ± 0.08	P = 0.80
Control variables			
Total number of neurons recorded	48.9 ± 1.3	47.5 ± 1.3	P = 0.44
Time spent in assembly field (min)	3.02 ± 0.19	3.23 ± 0.23	P = 0.49

Table S1. *(Refers to Figure 4)* Characteristics of early stabilized and gradually strengthened assembly patterns

With the exception of the correlation of member-pairs' PFS-scores, reported data are mean ± SEM and the P-values refer to unpaired two-sample *t*-tests for difference in mean. A neuron is considered to have a high contribution to an assembly pattern if its weight to that pattern is more than 2 standard deviations above the mean. Note that the average reactivation strength is significantly greater than zero for both the early stabilized ($t(81) = 4.16$, $P < 0.0001$, one-sample *t*-test) and the gradually strengthened ($t(56) = 3.57$, $P < 0.001$, one-sample *t*-test) assembly patterns, indicating that both sets of patterns are preferentially reactivated during the sleep/rest session following the exposure session compared to the baseline sleep/rest session recorded before. Also note that the average environment-specificity index is significantly greater than zero for both the early stabilized ($t(73) = 6.39$, $P < 0.0001$, one-sample *t*-test) and the gradually strengthened ($t(54) = 7.05$, $P < 0.0001$, one-sample *t*-test) assembly patterns, indicating that both sets of patterns are more similar to the patterns expressed during re-exposure to the same enclosure than to the patterns expressed in another enclosure. A single-neuron's place field similarity score is calculated as the Pearson correlation coefficient from the direct bin-wise comparison between its firing rate map in the novel enclosure and its firing rate map in the enclosure of the previous recording block on that day, whereby only spatial bins were included that were visited by the animal in both sessions (Kentros et al., 1998). The similar scores on this "single-neuron remapping measure" indicate that high contributing neurons of early stabilized patterns remapped to a similar degree as high contributing neurons of gradually strengthened patterns. In addition, remapping was assessed by measuring the extent to which the spatial overlap between the place fields of two neurons with high contribution to a given pattern detected in the novel enclosure was reminiscent of their spatial overlap in another enclosure previously explored. For this, the correlation of member-pairs' PFS-scores is calculated over all pairs of neurons with high contribution to the same pattern as the Pearson correlation coefficient between their place-field similarity (PFS) scores from both enclosures (McNamara et al., 2014). This measure is reported ± SE of the correlation coefficient and the P-value refers to the comparison of both correlations with a *z*-test after application of Fisher's *r* to *z* transform. The similar scores on this "neuron-pair remapping measure" indicate that there was no difference between early stabilized and gradually strengthened patterns in the extent to which pairs of neurons with high contribution to the same pattern already had overlapping place fields in the previous enclosure.

SUPPLEMENTAL EXPERIMENTAL PROCEDURES

Subjects

All animals used were male adult transgenic CamKIIa-Cre mice (4-7 months-old; Jackson Laboratories, B6.Cg-Tg(Camk2a-cre)T29-1Stl/J, stock number 005359; RRID: IMSR_JAX:005359). Animals had free access to water and food in a dedicated housing facility with a 12/12 h light/dark cycle. They shared a cage with their littermates until the injection surgery, after which they were housed alone. Surgical procedures were performed under deep anaesthesia using isoflurane (0.5-2 %) and oxygen (2 l/min), with analgesia (0.1 mg/kg vetergesic or 5 mg/kg metacam) provided before and after. All experiments involving animals were conducted according to the UK Animals (Scientific Procedures) Act 1986 under personal and project licenses issued by the Home Office following ethical review.

Viral vector injection for ArchT expression

The light-controlled, outward proton-pump ArchT (Han et al., 2011) was expressed in hippocampal principal neurons using a Cre-loxP approach (see **Figures 1A** and **S3B,C**). We injected a Cre-recombinase-dependent adeno-associated viral (AAV) vector containing ArchT-GFP (rAAV2-CAG-FLEX-ArchT-GFP; UNC Vector Core) in the dorsal CA1 of CamKII-Cre mice (Tsien et al., 1996) under stereotaxic control (Franklin and Paxinos, 2007). At four sites (coordinates: 1.7 / 2.3 mm posterior and ±1.7 mm lateral from bregma, and 1.1 mm ventral from the brain surface), 0.4 µl of the viral vector was injected at a rate less than 0.1 µl/min. Injections were performed with 5 µl calibrated glass micropipettes (Blaubrand), which were pulled and broken to have tip diameter ~17-20 µm.

Microdrive implantation

After two weeks of recovery mice were implanted with a custom-made microdrive. The drive was designed to bilaterally target the pyramidal layer of dorsal CA1, with for each hemisphere one optic fibre (230 µm diameter, Doris Lenses) surrounded by five independently moveable tetrodes. Both the distance from the optic fibre to each of the tetrodes and the distance between neighbouring tetrodes was 0.4 mm. Tetrodes were constructed by twisting together four insulated tungsten wires (12 µm diameter, California Fine Wire) and shortly heating them to bind them together in a single bundle. Each tetrode was attached to a 6 mm long M1.0 screw to enable their independent movement. The drive was implanted under stereotaxic control (coordinates for optic fibre tips: 2 mm posterior, ±1.7 mm lateral, 1.1 mm ventral). Tetrodes were placed ~500 µm dorsal to the tip of the optic fibres so that they were initially above the CA1 pyramidal layer. Following the implantation the exposed parts of the optic fibres and tetrodes were covered with paraffin wax, after which the drive was secured to the skull using dental cement. For extra stability, four stainless-steel anchor screws had first been inserted into the skull. Two of the anchor screws, which were inserted above the cerebellum, were attached to 50 µm tungsten wires (California Fine Wire) and served as ground and reference electrodes during the recordings. The placement of the tetrodes in dorsal CA1 was daily confirmed by the electrophysiological profile of the local field potential in the hippocampal ripple frequency band (Csicsvari et al., 1999, 2000; see **Figure S4A,C,D**), and at the end of the procedure by the anatomical verification of the tetrode tracks (see below and **Figure S4B**).

Recording procedures

After recovery from the implantation surgery, each animal was connected to the recording apparatus and familiarized with a 12 x 12 cm high-walled box containing home cage bedding (the “sleep box”) and with one of the open-field enclosures (the familiar enclosure) over a period of approximately seven days. During this period, tetrodes were gradually lowered to be positioned in the stratum oriens, above the CA1 pyramidal layer. On the morning of each recording day, tetrodes were lowered into the pyramidal cell layer in search of multi-unit spiking activity and sharp wave-ripple (SWR) events (see **Figure S4A**). Tetrodes were not moved for at least 2 h before recordings started. At the end of each recording day, tetrodes were raised back to the stratum oriens to avoid damaging the pyramidal layer overnight. Note that because tetrodes were daily moved in and out of the pyramidal cell layer, it is unlikely that all the cells recorded on a given day were the same as in other days, although this cannot be completely ruled out.

Each recording day, hippocampal network activity was continuously monitored as the animal completed two or three “recording blocks” (see **Figures 1B** and **S1A**). Each day started with a ~25 min period in the sleep box (“rest before”). For each recording block, the animal was then allowed to explore an open-field enclosure for ~25 min (“exposure”), followed by 1 h in the sleep box (“rest after”) before being allowed to again explore that enclosure for another ~25 min (“re-exposure”). The open-field enclosure was either the familiar enclosure, which the animal had repeatedly been exposed to before, or a novel enclosure the animal had never seen before. The open-field enclosures differed in shape and in the cue-cards that lined some of the walls, but they all had an area of ~0.25 m^2. All enclosures were placed in the same position within the same recording room and were surrounded by a black curtain to prevent the animal from using distal cues as a common reference frame for all

enclosures. In some of the recording blocks, the animal received optogenetic SWR silencing or optogenetic random silencing during the 1 h period in the sleep box between both exposures to the same enclosure (see below). The particular order of the “familiar” and “novel” recording blocks, as well as that of the optogenetic silencing and no silencing sleep/rest sessions, was varied between recording days. The present study includes a total of 93 recording blocks: 17 with the familiar enclosure without light delivery, 13 with the familiar enclosure with SWR silencing, 26 with a novel enclosure without light delivery, 24 with a novel enclosure with SWR silencing and 13 with a novel enclosure with random silencing.

Multichannel data acquisition and position tracking
The extracellular signals picked up by each electrode were buffered directly above the head of the animal and sent over individual wires to a dual-stage amplifier (Sensorium Inc.). After amplification (1000x) and band pass filtering (0.1 Hz to 5 kHz) the signals were digitized at 20 kHz and saved to a computer along with synchronization signals from the position tracking and laser stimulation. To track the location (and estimate the locomotion speed) of the animal, three LED lights (red, green and blue) were attached to the cable directly above the head of the animal and traced by an overhead color camera capturing 25 frames per second.

SWR detection and optogenetic silencing
A 561 nm diode-pumped solid-state laser (Laser 2000, Ringstead) was used to deliver light (~15 mW) to the dorsal CA1 pyramidal layer of CamKII::ArchT mice through two lengths of optic fiber and a rotary joint with splitter (Dorris Lenses). Light pulses were delivered either conditioned to the on line detection of SWR events or with randomly selected intervals. For the on line SWR detection (see also **Figure S3A**), one channel from the tetrode with the most pronounced ripples (“SWR detection electrode”) and one channel from a tetrode left in the cortex (“reference electrode”) were selected. The amplified and filtered electrophysiological signal from these two channels was sent to a computer with real-time kernel. To remove noise present on all tetrodes (e.g., muscle artefacts), the signal of the reference electrode was first subtracted from the signal of the SWR detection electrode. Then, every 5 ms, one copy of the last 20 ms of the differential signal was band-pass filtered (125-250 Hz, 19th order Butterworth filter), and another copy was convolved with a Morlet wavelet (160 Hz central frequency). If in the last 10 ms of this time-window the average power (root-mean-square) of the band-pass filtered signal was more than 4 SD above its baseline and the maximum of the wavelet-convoluted signal was more than 3 SD above its baseline, a SWR was said to be detected. For both the average power and for the maximum of the wavelet-convoluted signal, the baseline and SD were calculated using the first 25 sec of the recording session. Upon detection of a SWR, a 50 or 80 ms light pulse was triggered followed by a 30 ms refractory period during which no new light pulse could be triggered. To evaluate the accuracy of the on line SWR detection, SWRs were identified offline as previously described (McNamara et al., 2014). For the random silencing control experiment, the intervals between subsequent pulses were independently sampled from a uniform random distribution between 30 ms and X ms, whereby X was chosen for each animal to ensure the total number of random pulses to be equal to or higher than the average number of pulses delivered in the SWR silencing condition (number of pulses during 1 h sleep session: SWR silencing = 1939 ± 121, n = 37 recording blocks; random silencing = 2469 ± 169, n = 13). In one recording block, the optogenetic silencing triggered a very pronounced and unusual network response, as for example manifested by an abnormal high occurrence (4.6 Hz) of ripple-like events during the first 5 min of active exploration in the subsequent re-exposure session. As this was not observed on any other days (ripple occurrence rate is > 7.5 interquartile ranges above the second highest observed rate), this recording block was not included in this study.

Spike detection and unit isolation
For the offline detection of spikes, the recorded signals were first band-pass filtered (800 Hz to 5 kHz). Spikes were then detected based on the power (root-mean-square) of the filtered signal calculated in 0.2 ms sliding windows. Detected spikes of the individual electrodes were combined per tetrode. To isolate spikes putatively belonging to the same neuron, spike waveforms were first up-sampled to 40 kHz and aligned to their maximal through (Csicsvari et al., 1998). Principal component analysis (PCA) was applied to these waveforms ±0.5 ms from the through to extract the first three or four principal components per channel, such that each individual spike was represented by 12 waveform parameters. An automatic clustering program (KlustaKwik, http://klusta-team.github.io) was run on this principal component space and the resulting clusters were manually recombined and further isolated based on cloud shape in the principal component space, cross-channels spike waveforms, auto-correlation histograms and cross-correlation histograms (Harris et al., 2000). All sessions recorded on the same day were concatenated and clustered together. Each cluster used for further analysis showed throughout the entire recording day stable cross-channels spike waveforms, a clear refractory period in its auto-correlation histogram, well-defined cluster boundaries and an absence of refractory period in its cross-correlation histograms with the other clusters (McNamara et al., 2014). Yet, to further control for possible mistakes in unit isolation, we repeated the central analysis of this study after discarding all assembly patterns with two or more

high-contributing neurons recorded from the same tetrode (see **Figure S3F**). Moreover, for the neuron-pair analysis (see **Figure S3G**), only pairs of neurons that were recorded from different tetrodes were included. Hippocampal principal neurons were identified by the shape of their auto-correlation histogram, their firing rate and their spike waveform (Csicsvari et al., 1998).

Latency of CA1 principal neuron response to light pulses

The response-latencies of CA1 principal neurons to onset and termination of light pulses were calculated per sleep/rest session with SWR silencing. The time it took CA1 principal neurons to get silenced after onset of the light pulse was obtained by calculating the average firing rate time-locked to pulse onset in 1 ms bins. The principal neurons were deemed silenced when their average firing rate fell below 0.25 Hz. To calculate the time it took CA1 principal neurons to return to their baseline after termination of the pulse, the firing rate of the recorded principal neuron population was first calculated from 250 to 100 ms before pulse onset. The principal neurons were deemed to have returned to baseline when their average firing rate rose to this level.

Assembly pattern identification

Co-activation patterns of principal neurons were identified during the exposure session of each recording block (see **Figure 1B**). Epochs of active exploration (McNamara et al., 2014) were divided into 25 ms time bins and for every principal neuron its number of spikes in each bin was counted. In order not to bias the identification of assembly patterns towards spike trains of neurons with higher firing rates, the binned spike counts were normalized for each neuron by a *z*-score transform:

$$z_{i,b} = \frac{x_{i,b} - \mu_{x_i}}{\sigma_{x_i}}$$

where $x_{i,b}$ was the spike count of neuron i in bin b, and μ_{x_i} and σ_{x_i} were respectively the mean and standard deviation of neuron i's spike counts across bins. This ensured that the normalised activity of each principal neuron had null mean rate and unitary variance. With the number of principal neurons denoted by n and the number of time bins by B, let $\mathbf{Z}$ be the $n \times B$ binned and *z*-scored spike count matrix with element (i, b) equal to $z_{i,b}$. From this matrix $\mathbf{Z}$, *assembly patterns* were identified in a two-step procedure (Lopes-dos-Santos et al., 2013; Trouche et al., 2016):

Determine number of significant patterns: a principal component analysis (PCA) was first applied to matrix $\mathbf{Z}$:

$$\sum_{j=1}^{n} \lambda_j \boldsymbol{p}_j \boldsymbol{p}_j^{\mathrm{T}} = \frac{1}{n} \mathbf{Z}\mathbf{Z}^{\mathrm{T}}$$

where $\boldsymbol{p}_j$ is the jth principal component with corresponding eigenvalue λ_j (for $j = 1, \ldots, n$). Note that $\frac{1}{n}\mathbf{Z}\mathbf{Z}^{\mathrm{T}}$ is the correlation matrix of $\mathbf{Z}$. To estimate the number of significant patterns embedded within the data, we used the Marčenko-Pastur law (Marčenko and Pastur, 1967; Götze et al., 2004). This law states that for a $n \times B$ matrix whose elements are independent and identically distributed random variables with zero mean and unit variance, all eigenvalues are asymptotically (i.e., when $n, B \to \infty$ such that B/n converges to a finite positive value) bounded to the interval $\left[\left(1 - \sqrt{n/B}\right)^2, \left(1 + \sqrt{n/B}\right)^2\right]$. This suggests that if the firing activity of the neurons is independent from each other, then none of the eigenvalues is expected to exceed $\lambda_{\max} = \left(1 + \sqrt{n/B}\right)^2$. Using simulated spike trains, this was indeed shown to be the case even for values of n and B considerably smaller than in our data-set (Peyrache et al., 2010; Lopes-dos-Santos et al., 2011, 2013). An eigenvalue above $\lambda_{\max}$ thus indicates that the pattern given by the corresponding principal component captures more correlation than any pattern would be expected to capture if the firing activity of all neurons was independent of each other. The number of eigenvalues above $\lambda_{\max}$ (denoted by N_A) therefore represents the number of distinct significant patterns.

Identify assembly pattern composition: the first N_A principal components each capture a significant amount of correlation between the firing activities of the neurons. However, principal components are restricted to be orthogonal to each other, while cell assemblies do not need to be (e.g., they can contain overlapping neurons). Principal components are also extracted from the data sequentially, which usually causes the first principal component to seemingly be a mixture of multiple assemblies (Laubach et al., 1999; Lopes-dos-Santos et al., 2011). Moreover, a PCA is solely based on pairwise correlations, but higher-order correlations could also inform assembly identification. To avoid these caveats, we therefore used an independent component analysis (ICA) to identify the patterns. An ICA extracts patterns such that the linear projections of the data onto these patterns are as independent from each other as possible. However, an ICA directly applied to matrix $\mathbf{Z}$ without

prior dimension reduction would extract as many patterns as there are neurons, which leads to spurious results (Lopes-dos-Santos et al., 2013). To restrict the number of patterns identified by ICA to N_A, the data is first projected onto the subspace spanned by the first N_A principal components:

$$\mathbf{Z}_{\mathrm{PROJ}} = \mathbf{P}_{\mathrm{SIGN}}{}^{\mathrm{T}}\mathbf{Z}$$

where $\mathbf{P}_{\mathrm{SIGN}}$ is the $n \times N_A$ matrix with the first N_A principal components as columns. An ICA was then applied to the matrix $\mathbf{Z}_{\mathrm{PROJ}}$. That is, a $N_A \times N_A$ un-mixing matrix $\mathbf{W}$ was found such that the rows of the matrix $\mathbf{Y} = \mathbf{W}^{\mathrm{T}}\mathbf{Z}_{\mathrm{PROJ}}$ are as independent as possible. This optimization-problem was solved using the fastICA algorithm (Hyvärinen, 1999) implemented in R (fastICA-package: Marchini et al., 2013). The resulting un-mixing matrix $\mathbf{W}$ was then expressed in the original basis spanned by all the neurons:

$$\mathbf{V} = \mathbf{P}_{\mathrm{SIGN}}\mathbf{W}$$

where the columns of $\mathbf{V}$ (i.e., $\boldsymbol{v}_1$, …, $\boldsymbol{v}_{N_A}$) are the weight vectors of the *assembly patterns*. As both the sign and the scale of the ICA output is arbitrary, all weight vectors were scaled to unit length (i.e., $\sum_{i=1}^{n} \boldsymbol{v}_k{}^2(i) = 1$ for $k = 1, \dots, N_A$) and their sign set such that the highest absolute weight of each assembly pattern was positive. Note that the detection of assembly patterns is solely based on neuronal co-activations within 25 ms time bins; the particular sequence of activations within these co-activation events is not considered here. Also note that for a co-activation pattern to be detected as significant, it needs to be active in many different time bins.

In the exposure sessions of the 93 recording blocks, we detected a total of 521 assembly patterns: 108 in blocks with the familiar enclosure without light delivery, 78 in blocks with the familiar enclosure followed by SWR silencing, 139 in blocks with a novel enclosure without light delivery, 136 in blocks with a novel enclosure followed by SWR silencing and 60 in blocks with a novel enclosure followed by random silencing.

Most of the detected assembly patterns consisted of a few neurons with high weights and a large group of neurons with weights around zero (e.g., see **Figures 1C** and **S2A**). For each assembly pattern it would therefore be possible to define a corresponding "cell assembly" as those neurons whose weight exceeds the mean weight by two standard deviation (*member neurons*; with both mean and standard deviation calculated from only the weights of that pattern). Note that in this study, this definition of cell assemblies is only used for an intuitive way of interpreting and discussing the presented results. Importantly, all analyses were performed directly on the assembly patterns themselves (i.e., using the weight vectors formed by the contribution of all recorded principal neurons). The sparsity of an assembly pattern (see **Table S1**), which reflects to what extent the weight vector of a pattern is dominated by a small group of neurons, was calculated as

$$1 - \frac{\sqrt{n} - \sum|v_i|}{\sqrt{n} - 1}$$

where n is the length of the weight vector (i.e., the number of principal neurons recorded that day) and v_i is the weight vector's i^{th} element (i.e., the contribution of neuron i to the pattern).

Environment specificity

To assess the environment specificity of assembly patterns, we compared each pattern detected during the first exposure to an enclosure with the set of patterns detected during re-exposure to the same enclosure and with the set of patterns detected during exposure to another enclosure of a different recording block. The similarity of two assembly patterns was quantified by a *similarity index* equal to the absolute value of the inner-product of their weight vectors (Almeida-Filho et al., 2014). A pattern's *environment-specificity index* was then defined as its maximum similarity index with any of the patterns detected during re-exposure minus its maximum similarity index with any of the patterns detected in the other enclosure.

Tracking expression of assembly patterns over time

In order to evaluate the reactivation and reinstatement strength of the assembly patterns identified during the first exposure session, we tracked the expression of each pattern over time:

$$R_k(t) = \boldsymbol{z}(t)^{\mathrm{T}}\mathbf{P}_k\boldsymbol{z}(t)$$

where $R_k(t)$ is the *expression strength* of assembly pattern k at time t and $\boldsymbol{z}(t)$ is a smooth vector-function containing for each neuron its *z*-scored instantaneous firing rate. $\mathbf{P}_k$ is the projection matrix of pattern k and was constructed from the outer product of its weight vector $\boldsymbol{v}_k$. The diagonal entries of this projection matrix were set to zero to prevent high expression strength caused by the isolated activity of a single neuron with high weight to that pattern (Peyrache et al., 2009, 2010, Lopes-dos-Santos et al., 2011, 2013). With this approach, only co-firing of neurons can contribute towards the expression of an assembly pattern. To increase the temporal

resolution beyond the bin-size used to identify the assembly patterns, $\boldsymbol{z}(t)$ was obtained by convolving the spike train of each neuron with a kernel-function (Dupret et al., 2013; Lopes-dos-Santos et al., 2013) after which the resulting smooth curve was normalized by a *z*-score transform. As smoothing function, a Gaussian kernel was chosen with standard deviation $w/\sqrt{12}$, so that the kernel had the same standard deviation as fixed time bins of w ms (Kruskal et al., 2007). The value w determines the width of the "integration window" in which spikes are still considered to be coincident. We set w to 25 ms to match the bin-size used to identify the patterns. The resulting expression strength time-courses showed a low baseline with sparse, transient peaks (see **Figure 1C**). These peaks correspond to co-activations (within 25 ms) of at least two neurons with high weight in that pattern, whereby the magnitude of these peaks is governed by both (1) the weights of the active neurons in that pattern and (2) the number of spikes discharged by each neuron within the window. Note that the scale of the expression strength can be interpreted as a "projected *z*-score". *Assembly pattern activations* were defined as peaks in the expression strength exceeding $R_{\mathrm{THRES}} = 5$. During the exposure session in which the patterns were detected, this threshold resulted in a mean assembly pattern activation rate of 0.95 ± 0.02 Hz (*n* = 521 assembly patterns). Note that each detected assembly pattern thus corresponded to many activations.

Assembly pattern activation maps
The time stamps of these assembly pattern activations during active exploration in the first exposure session were used to generate *assembly maps* (e.g., **Figures 1C** and **4B**). For this, the horizontal plane of the recording room was divided into spatial bins of approximately 2 x 2 cm. Activation count maps (number of assembly pattern activations per spatial bin) and an occupancy map (time spent by the animal in each spatial bin) were generated, and all maps were spatially smoothed by convolution with a Gaussian kernel with standard deviation of one bin width. Smoothed activation count maps were then normalized by dividing them by the smoothed occupancy map to generate the spatial assembly maps.
The coherence and sparsity of an assembly map (see **Table S1**) were calculated from the unsmoothed maps. Coherence, which reflects the similarity of the assembly pattern activation rate in adjacent bins, was calculated as Fisher's *r* to *z* transform of the Pearson correlation (across all visited bins) between the rate in a bin and the average rate of its eight nearest neighbours (Muller and Kubie, 1989). Sparsity, which reflects how tightly concentrated in space the activity of an assembly pattern is, was calculated as $(\sum P_i R_i)^2 / \sum P_i {R_i}^2$, where P_i is the propability of the animal occupying bin i and R_i is the assembly pattern activation rate in bin i (Skaggs et al., 1996). A pattern's assembly field (see **Table S1**) was defined as those bins with an assembly pattern activation rate above its "field threshold", which was calculated as the rate of the bin with the highest rate minus 40 % of the difference in rate between the bins with the highest and lowest rate.

Reactivation and reinstatement strength
To compare offline reactivation across assembly patterns, regardless of their difference in baseline strength (e.g., see **Figure S1F**), the *reactivation strength* of an assembly pattern identified during the exposure session was defined as its average expression strength during the rest after minus its average expression strength during the rest before. Note that a null score for reactivation strength indicates that a pattern is not reactivated (i.e., equal strength during rest after as during rest before), while the more positive the stronger the reactivation of that pattern. The *reinstatement strength* was similarly defined as its average expression strength during re-exposure minus its average expression strength during the first exposure. Note that a null score for reinstatement strength indicates that a pattern is "perfectly" reinstated (i.e., no loss in strength from initial exposure to re-exposure), while the more negative the worse the reinstatement of that pattern (see the legend of **Figure S3E** for further discussion of this measure).

Gradually strengthened and early stabilized assembly patterns
Assembly patterns identified during the first exposure to a novel enclosure were divided into two sets based on the time-course of their expression strength. For each pattern, a linear trend model (including intercept) was fitted to its expression strength during the exposure session calculated in 1 sec bins. Patterns with a significant positive slope were defined as *gradually strengthened* and those without significant positive slope as *early stabilized*. Note that these two sets of patterns were defined solely based on their strengthening dynamics during the initial exposure session, and that we then show that both sets later differ in their sensitivity to SWR disruption. Further note that this division of patterns into two sets was only used for the analyses corresponding to **Figure 4**; all other analyses were always based on all detected assembly patterns. In **Figure 4A**, for visualization-purposes only, the individual expression strength time-courses were smoothed with a Gaussian kernel (SD = 5 sec) before calculating the presented average and SEM time-courses. One gradually strengthened assembly pattern detected in a novel enclosure had both an unusual high reactivation and reinstatement strength. This pattern would be very influential for the reinstatement-vs-reactivation correlation presented in **Figures 2** and **4C**. We therefore left the data-point corresponding to this pattern ([0.62, 0.14]) out from these graphs; its

inclusion would further increase the correlation for novel-blocks to $r = 0.41$ and the correlation for gradually strengthened patterns to $r = 0.57$.

Neuron-pair and single-neuron measures

For each pair of principal neurons, the *co-firing coefficient* (see **Figure 1D**) was calculated as the Pearson correlation coefficient between the binned (25 ms time bins) spike counts of the two neurons forming that pair (McNamara et al., 2014). To calculate the *place-field similarity* (PFS; see **Figures 1E**, **S1C** and **S3G**) scores between two neurons in a given exploratory session, spatial rate maps were first generated for both neurons in a way similar to the assembly maps, with the assembly pattern activations replaced by the neuron's spike train. The PFS was then defined as the Pearson correlation coefficient from the direct comparison of the spatial bins between the spatial rate maps of both neurons (McNamara et al., 2014). For the single-neuron analyses (see **Figures S1D** and **S3H**), a given neuron's place field *similarity score* between two exploratory sessions was calculated as the Pearson correlation coefficient from the direct bin-wise comparison between that neuron's spatial rate maps from both sessions, whereby only spatial bins were included that were visited by the animal in both sessions (Kentros et al., 1998). For all neuron-pair and single-neuron analyses, only principal neurons with at least 100 spikes discharged during active exploration in each session considered were included. In addition, for the neuron-pair analyses, only pairs of neurons recorded from different tetrodes were included.

Anatomical confirmation of tetrode placement

At the completion of the experiment, all implanted mice were deeply anesthetized with isoflurane/pentobarbital and transcardially perfused with 0.1 M phosphate-buffered saline (PBS) followed by cold 4 % paraformaldehyde (PFA) dissolved in PBS (McNamara et al., 2014). Implanted tetrodes were kept in the brain for at least 4 days of post-perfusion fixation in 4 % PFA before being removed and the brain being extracted. Brains were then kept in 4 % PFA for at least 24 h before slicing. In order to visualise tetrode tracks, brains were sliced into 100 µm thick coronal sections and stained using osmium. To do so, brain sections were rinsed extensively in 0.1 M phosphate buffer (PB) before being immersed in 4 % PFA with 0.05 % glutaraldehyde for 15 min. Sections were then rinsed three times in PB for 10 min and immersed in PB with 0.5 % osmium for 1 h. Sections were rinsed again in PB and dehydrated by successive immersion in alcohol and propylenoxid. Sections were finally mounted on slides and cover-slipped. The images were acquired on a microscope (Axio Imager M2; Zeiss) with 5x/0.1 NA objective and mounted color camera (Retiga 2000R; QImaging) under bright-field illumination. They were converted to greyscale, inverted and contrasted to best illustrate the layers of the hippocampus and the tetrode tracks.

Verification of ArchT-GFP expression

To confirm the injection-site and specificity of the viral vector, 6 weeks after the injection-surgery CamKII::ArchT mice were deeply anesthetized with isoflurane/pentobarbital and transcardially perfused with PBS followed by cold 4 % PFA and 0.1 % glutaraldehyde dissolved in PBS. The brains were extracted and sliced into 50 µm thick coronal sections. The sections were rinsed extensively in PBS with 0.25 % Triton X-100 (PBS-T) and blocked for 1 h at room temperature in PBS-T with 10 % normal donkey serum (NDS). Sections were then incubated at 4 °C for 72 h with primary antibodies (chicken anti-GFP, 1:1000, Aves Labs, cat# GFP-1020, RRID: AB_10000240 and rabbit anti-Wfs1, 1:500, Proteintech Group, cat# 11558-1-AP, RRID: AB_2216046) diluted in PBS-T with 3 % NDS ("blocking solution"). After that, sections were rinsed three times for 15 min in PBS-T and incubated for 4 h at room temperature in secondary antibodies (donkey anti-chicken Alexa Fluor 488, 1:500, Jackson ImmunoResearch, cat# 703-545-155, RRID: AB_2340375 and donkey anti-rabbit Cy3, 1:1000, Jackson ImmunoResearch, cat# 711-165-152, RRID: AB_2307443) diluted in the blocking solution. This step was followed by three rinses for 15 min in PBS. Sections were then incubated for 1 min with 4',6-diamidino-2-phenylindole (DAPI; 0.5 µg/ml, Sigma-Aldrich, cat# D8417) diluted in PBS to label cell nuclei, before undergoing three additional rinse steps of 10 min each in PBS. Sections were finally mounted on slides, cover-slipped with Vectashield mounting medium (Vector Laboratories) and stored at 4 °C. Images were acquired using an epifluorescence microscope (Axio Imager M2; Zeiss) and Stereo Investigator software (Virtual Tissue 2D module; MBF Bioscience). Cell counting was performed on *z*-stacks obtained with a 40x/1.3 NA objective using Stereo Investigator and ImageJ (http://rsb.info.nih.gov/) software and was conducted from every fourth coronal section containing the dorsal CA1 hippocampus (25 cells per section, 4 sections per mouse, 2 mice). The percentage of principal neurons transfected with ArchT-GFP was evaluated by the percentage of cells immunopositive for Wolfram syndrome 1 (Wfs1), a marker specific for CA1 principal neurons (Lein et al., 2007; Valero et al., 2015; Cembrowski et al., 2016), that were immunopositive for the GFP reporter.

Statistical analysis

The means of two sets of observations were compared with a two-sample *t*-test or, if they were obtained from the same set of assembly patterns, with a paired *t*-test. To test for a differential effect of SWR silencing, a 2-way

ANOVA was performed. Whether the average reactivation strength or the average environment-specificity index of a set of assembly patterns differed from zero was tested with a one-sample *t*-test. Testing whether a Pearson correlation coefficient (r) differed from zero was done with a standard *t*-test, with the standard error calculated as the square root of $(1 - r^2)/(n - 2)$, where n is the number of paired observations. Comparison of two Pearson correlation coefficients was done with a *z*-test, after application of Fisher's r to z transform (p. 386-387; Zar, 1999). Whether the proportion of patterns with higher expression strength during rest after than during rest before differed from the 50 % chance level was tested with a one-sample *z*-test for proportions. The linear regression models used contained an intercept and slope and were estimated using ordinary least squares. Whether the fitted slope differed from zero was tested with a *t*-test. All *t*-tests and *z*-tests were performed two-sided. Error bars are ±1 standard error of the mean (SEM) or ±1 standard error (SE) of the correlation coefficient. Reported group data are mean ± SEM, unless stated otherwise.

SUPPLEMENTAL REFERENCES

Almeida-Filho, D.G., Lopes-dos-Santos, V., Vasconcelos, N. a P., Miranda, J.G.V., Tort, A.B.L., and Ribeiro, S. (2014). An investigation of Hebbian phase sequences as assembly graphs. Front. Neural Circuits *8*, 34.

Berke, J.D., Hetrick, V., Breck, J., and Greene, R.W. (2008). Transient 23–30 Hz oscillations in mouse hippocampus during exploration of novel environments. Hippocampus *18*, 519–529.

Cembrowski, M.S., Wang, L., Sugino, K., Shields, B.C., and Spruston, N. (2016). Hipposeq: a comprehensive RNA-seq database of gene expression in hippocampal principal neurons. eLife *5*, e14997.

Csicsvari, J., Hirase, H., Czurko, A., and Buzsáki, G. (1998). Reliability and State Dependence of Pyramidal Cell–Interneuron Synapses in the Hippocampus. Neuron *21*, 179–189.

Csicsvari, J., Hirase, H., Czurko, A., Mamiya, A., and Buzsáki, G. (1999). Fast network oscillations in the hippocampal CA1 region of the behaving rat. J. Neurosci. *19*, 1–4.

Csicsvari, J., Hirase, H., Mamiya, A., and Buzsáki, G. (2000). Ensemble Patterns of Hippocampal CA3-CA1 Neurons during Sharp Wave–Associated Population Events. Neuron *28*, 585–594.

Dupret, D., O'Neill, J., and Csicsvari, J. (2013). Dynamic reconfiguration of hippocampal interneuron circuits during spatial learning. Neuron *78*, 166–80.

Franklin, K.B.J., and Paxinos, G. (2007). Mouse Brain in Stereotaxic Coordinates (Academic Press).

Götze, F., Tikhomirov, A., and others (2004). Rate of convergence in probability to the Marchenko-Pastur law. Bernoulli *10*, 503–548.

Han, X., Chow, B.Y., Zhou, H., Klapoetke, N.C., Chuong, A., Rajimehr, R., Yang, A., Baratta, M.V., Winkle, J., Desimone, R., et al. (2011). A high-light sensitivity optical neural silencer: development and application to optogenetic control of non-human primate cortex. Front. Syst. Neurosci. *5*, 18.

Harris, K.D., Henze, D.A., Csicsvari, J., Hirase, H., and Buzsáki, G. (2000). Accuracy of tetrode spike separation as determined by simultaneous intracellular and extracellular measurements. J. Neurophysiol. *84*, 401–414.

Hyvärinen, A. (1999). Fast and robust fixed-point algorithms for independent component analysis. IEEE Trans. Neural Netw. *10*, 626–634.

Jackson, J.C., Johnson, A., and Redish, A.D. (2006). Hippocampal Sharp Waves and Reactivation during Awake States Depend on Repeated Sequential Experience. J. Neurosci. *26*, 12415–12426.

Kentros, C., Hargreaves, E., Hawkins, R.D., Kandel, E.R., Shapiro, M., and Muller, R.V. (1998). Abolition of Long-Term Stability of New Hippocampal Place Cell Maps by NMDA Receptor Blockade. Science *280*, 2121–2126.

Kruskal, P.B., Stanis, J.J., McNaughton, B.L., and Thomas, P.J. (2007). A binless correlation measure reduces the variability of memory reactivation estimates. Stat. Med. *26*, 3997–4008.

Laubach, M., Shuler, M., and Nicolelis, M.A.L. (1999). Independent component analyses for quantifying neuronal ensemble interactions. J. Neurosci. Methods *94*, 141–154.

Lein, E.S., Hawrylycz, M.J., Ao, N., Ayres, M., Bensinger, A., Bernard, A., Boe, A.F., Boguski, M.S., Brockway, K.S., Byrnes, E.J., et al. (2007). Genome-wide atlas of gene expression in the adult mouse brain. Nature *445*, 168–176.

Lopes-dos-Santos, V., Conde-Ocazionez, S., Nicolelis, M.A.L., Ribeiro, S.T., and Tort, A.B.L. (2011). Neuronal assembly detection and cell membership specification by principal component analysis. PloS One *6*, e20996.

Marčenko, V.A., and Pastur, L.A. (1967). Distribution of eigenvalues for some sets of random matrices. Sb. Math. *1*, 457–483.

Marchini, J.L., Heaton, C., and Ripley, B.D. (2013). fastICA: FastICA Algorithms to perform ICA and Projection Pursuit.

Muller, R.U., and Kubie, J.L. (1989). The firing of hippocampal place cells predicts the future position of freely moving rats. J. Neurosci. *9*, 4101–4110.

Peyrache, A., Khamassi, M., Benchenane, K., Wiener, S.I., and Battaglia, F.P. (2009). Replay of rule-learning related neural patterns in the prefrontal cortex during sleep. Nat. Neurosci. *12*, 919–26.

Peyrache, A., Benchenane, K., Khamassi, M., Wiener, S.I., and Battaglia, F.P. (2010). Principal component analysis of ensemble recordings reveals cell assemblies at high temporal resolution. J. Comput. Neurosci. *29*, 309–25.

Skaggs, W.E., McNaughton, B.L., Wilson, M.A., and Barnes, C.A. (1996). Theta phase precession in hippocampal neuronal populations and the compression of temporal sequences. Hippocampus *6*, 149–172.

Trouche, S., Perestenko, P.V., van de Ven, G.M., Bratley, C.T., McNamara, C.G., Campo-Urriza, N., Black, S.L., Reijmers, L.G., and Dupret, D. (2016). Recoding a cocaine-place memory engram to a neutral engram in the hippocampus. Nat. Neurosci. *19*, 564–567.

Tsien, J.Z., Chen, D.F., Gerber, D., Tom, C., Mercer, E.H., Anderson, D.J., Mayford, M., Kandel, E.R., and Tonegawa, S. (1996). Subregion- and Cell Type–Restricted Gene Knockout in Mouse Brain. Cell *87*, 1317–1326.

Valero, M., Cid, E., Averkin, R.G., Aguilar, J., Sanchez-Aguilera, A., Viney, T.J., Gomez-Dominguez, D., Bellistri, E., and de la Prida, L.M. (2015). Determinants of different deep and superficial CA1 pyramidal cell dynamics during sharp-wave ripples. Nat. Neurosci. *18*, 1281–1290.

Zar, J.H. (1999). Biostatistical Analysis (Upper Saddle River, New Jerset, USA: Prentice Hall International, Inc.).
